# Supplementary material for: Breaking barriers for TB elimination: A novel community-led strategy revolutionizing tuberculosis case finding and treatment support in Senapati District Manipur-A quasi-experimental pre-post study protocol
Source: PLoS One. 2025 Jul 3;20(7):e0326324. doi: 10.1371/journal.pone.0326324 (PMC12225811; doi:10.1371/journal.pone.0326324)
Supplement: S1 File — Study tools and questionnaires used for data collection. Includes: Annexure I – Presumptive TB Case Referral Slip. Annexure II – TB Knowledge, Attitude, and Practice Questionnaire. Annexure III – Household Enumeration Questionnaire. Annexure IV – First Round TB Symptom Screening Tool. Annexure V – Second Round TB Symptom Screening Tool. Annexure VI – Part 1: Cost Identification Post-Treatment Initiation. Annexure VII – Parts 2 & 3: Cost Identification at End of Intensive Phase & End of Treatment. (DOCX) [file pone.0326324.s001.docx]

**Annexure-I**

**Presumptive TB case referral slip**

| Name of the volunteer / MTDV Lab technician / FS : | | |
| --- | --- | --- |
| Volunteer’s village: | Taluka: | Date: |
| Name of the chest symptomatic person (Full name) | | Age / Gender: M/F/TG |
| Full address of the symptomatic person | | Mobile number |
| Symptoms of the person (Circle the observed symptoms or told by the symptomatic person)   1. *Persistent cough for ≥2 weeks* 2. *Fever for ≥2 weeks* 3. *Loss of appetite* 4. *Night sweats for more than 2 weeks or unintentional weight loss (loss of 4.5kg or >5kg of the usual body weight over a period of 6-12 months)* 5. *Presence of blood in sputum any time during the last 6 months* 6. *Chest pain in last one month* | | |
| Referred to where?   1. Primary health centre 2. [Community Health Centre](https://vikaspedia.in/health/health-directory/rural-health-care-system-in-india#section4) 3. District Tuberculosis Centre 4. District hospital | Name of the health facility referred | |
| Signature of the volunteer | Any other details | |

**Annexure-II**

**TB Knowledge, attitude and practice questionnaire**

| **Section 1: Socio-demographic information** | | | | |
| --- | --- | --- | --- | --- |
| **Sl . No.** | **Questions** | **Response** | **Remarks** | |
|  | What is your age in years? | Years |  | |
|  | What is your sex? | 1. Male 2. Female 3. Other |  | |
|  | What is your marital status? | 1. Married 2. Unmarried 3. Divorced/separated 4. Widow/widower |  | |
|  | What is your highest level of education you have completed? | 1. Illiterate 2. No formal school but can read and write 3. Upto primary level 4. Upto secondary level 5. Upto graduation/degree level 6. Master level and above |  | |
|  | What is your occupation?  **(multiple answers)** | 1. House wife 2. Student 3. Own cultivation 4. Agriculture labourer 5. Small business/petti/ tea shop 6. Livestock 7. Private 8. Government 9. Other (Specify)………………………………………….. |  | |
|  | What is your monthly income (INR)? | 1. Up to 10000 2. 10001-20000 3. 20001-30000 4. 30001-40000 5. 40001-50000 6. 50001 and above |  | |
|  | Do you belong to the schedule tribe? | Yes No  If yes, specify………………. If no, specify......................... |  | |
| **Section 2: Knowledge on TB** | | | | |
| **Sl. No** | **Questions** | **Response** | **Remarks** | |
|  | Have you heard of TB?(use local term for TB) | 1. Yes 2. No | If no, end the survey | |
|  | Where did you first hear of TB? **(tick all that applies)** | 1. Newspapers/magazines 2. Radio 3. TV 4. Brochures, posters and other printed materials 5. Health workers 6. Family, friends, neighbours and colleagues 7. Religious leaders 8. Teachers 9. Social media 10. Present study team |  | |
|  | What causes TB? | 1. Virus 2. Bacteria 3. Fungus 4. protozoa 5. supernatural 6. don’t know |  | |
|  | What are the symptoms of lung TB?  **(Tick multiple answers)** | 1. Cough for more than 2 weeks 2. Fever 3. Loss of Weight 4. Blood in sputum 5. Chest pain 6. Loss of appetite 7. Fatigue 8. Abdominal pain 9. Rash 10. Others 11. Don’t know |  | |
|  | How is TB transmitted? | 1. Through handshakes 2. Through the air when a person with TB coughs or sneezes 3. Through sharing dishes 4. Through eating from the same plate 5. Through touching items in public places (doorknobs, handles in transportation, etc.) 6. Do not know. 7. Others …………………………………………………. |  | |
|  | How can you prevent TB? | 1. Avoid shaking hands 2. Covering mouth and nose when coughing or sneezing 3. Avoid sharing dishes 4. Washing hands after touching items in public places 5. Closing windows at home 6. Through good nutrition 7. By praying 8. Do not know |  | |
|  | What is the most common method of lung TB diagnosis? | 1. Blood test 2. Urine and stool test 3. Chest X-ray 4. Sputum test 5. Don’t know |  | |
|  | Is TB vaccine currently available? | 1. Yes 2. No 3. Don’t know |  | |
|  | How long does it take to treat TB? | 1. 1- 2 weeks 2. 1-2 month 3. 6 -8 months 4. 1-2 years 5. Don’t know |  | |
|  | Who can be infected with TB? | 1. Anybody 2. Only poor people 3. Only homeless people 4. Only alcoholics 5. Only drug users 6. Only people living with HIV/AIDS 7. Only people who have been in prison 8. Don’t know |  | |
|  | Can TB affect organs other than the lungs? | 1. Yes 2. No 3. Don’t know |  | |
|  | Should the TB patient discontinue drugs once he feels well? | 1. Yes 2. No 3. Don’t know |  | |
|  | What happens if a patient discontinues TB drugs? | 1. Patient gets better 2. Patient develops drug-resistant TB 3. Don’t know |  | |
|  | What could increase the risks of getting TB?  **(tick multiple answers)** | 1. Smoking 2. Drinking alcohol 3. Malnutrition 4. HIV 5. Diabetes 6. Don’t know |  | |
|  | Can a person who was previously treated for TB get infected again? | 1. Yes 2. No 3. Don’t know |  | |
| **Section 3: TB attitudes** | | | | |
|  | How serious is tuberculosis in your opinion? | 1. Very serious 2. Somewhat serious 3. Not very serious 4. Don’t know |  | |
|  | Is Tuberculosis a public health problem in India? | 1. Yes 2. No 3. Maybe 4. Don’t know |  | |
|  | Do you think you can get TB? | 1. Yes 2. Sometime 3. No 4. Don’t know |  | |
|  | I feel compassion and desire to help TB-infected persons | 1. Strongly disagree 2. Disagree 3. Neutral 4. Agree 5. Strongly agree |  | |
|  | I feel compassion but I tend to stay away from TB-infected persons | 1. Strongly disagree 2. Disagree 3. Neutral 4. Agree 5. Strongly agree |  | |
|  | I fear TB patients because they may infect me | 1. Strongly disagree 2. Disagree 3. Neutral 4. Agree 5. Strongly agree |  | |
|  | I will be embarrassed if I am told I have TB | 1. Strongly disagree 2. Disagree 3. Neutral 4. Agree 5. Strongly agree |  | |
|  | I will be sad and hopeless if I am told I have TB | 1. Strongly disagree 2. Disagree 3. Neutral 4. Agree 5. Strongly agree |  | |
|  | I will be ashamed if I am told that I have TB | 1. Strongly disagree 2. Disagree 3. Neutral 4. Agree 5. Strongly agree |  | |
|  | In my community, most people usually reject a person who has TB | 1. Strongly disagree 2. Disagree 3. Neutral 4. Agree 5. Strongly agree |  | |
|  | In my community, most people are friendly to TB patients but try to avoid them. | 1. Strongly disagree 2. Disagree 3. Neutral 4. Agree 5. Strongly agree |  | |
| **Section 4: Practice on TB** | | | | |
|  | Who would you first talk about your illness if you had TB symptoms?  **(Please tick all that apply)** | 1. Doctors or other medical workers 2. Spouse 3. Parents 4. Children 5. Other family members 6. Close friend 7. No one 8. Others: | |  |
|  | Where would you go if you had TB symptoms? | 1. Hospital 2. Medical shops/pharmacy 3. Traditional healer 4. Others: | |  |
|  | At what point of time would you go to health facility if you had TB symptom | 1. When treatment on my own does not work 2. When symptom last for 1-2 weeks 3. When symptom last for 3-4 weeks 4. As soon as I realize that my symptom might be related to TB 5. I will not go to the Doctors | |  |
|  | If you would not go to the health facility, what is the reason?  **(Please check all that apply)** | 1. Not sure where to go 2. Cost 3. Difficulties with transportation/distance to a clinic 4. Do not trust medical workers 5. Do not like the attitude of medical workers 6. Cannot leave work (overlapping work hours with medical facility working hours) 7. Do not want to find out that something is really wrong 8. Other (please explain) | |  |

**Annexure-III**

**Household enumeration questionnaire**

| **Sl. No** | **Household enumeration Questionnaire** | | | | | | |  |
| --- | --- | --- | --- | --- | --- | --- | --- | --- |
| 1 | Name of the block | | | |  | | |  |
| 2 | Name of the village | | | |  | | |  |
| 3 | Village No. | | | |  | | |  |
| 4 | Household No. | | | |  | | |  |
| 5 | Data Collector Name | | | |  | | |  |
| 6 | Data Collector Phone No. | | | |  | | |  |
| 7 | **a. Name of HH member** | **b. Age** | **c. Gender**   1. Male 2. Female 3. Other | **d. Education level**   1. Illiterate 2. No formal school but can read and write 3. Upto primary level 4. Upto secondary level 5. Upto graduation/degree level 6. Master level and above | **e. Head of HH relationship**   1. Head of HH 2. Spouse 3. Child 4. Relative 5. Other | **f. Occupations**   1. House wife 2. Student 3. Own cultivation 4. Agriculture labourer 5. Small business/petti/ tea shop 6. Livestock 7. Private 8. Government 9. Other   **(Multiple response)** | **g. Income per month (in Rs.)** | **h. What are the common health problems you have in the last 5 years?**  (Mention their symptom) |
|  |  |  |  |  |  |  |  |  |
|  |  |  |  |  |  |  |  |  |
|  |  |  |  |  |  |  |  |  |
|  |  |  |  |  |  |  |  |  |
|  |  |  |  |  |  |  |  |  |
|  |  |  |  |  |  |  |  |  |
|  |  |  |  |  |  |  |  |  |
|  |  |  |  |  |  |  |  |  |
|  |  |  |  |  |  |  |  |  |

**Annexure –IV**

**First Round TB Symptom Screening Tool**

| **Section:1 Socio Demographic profile** | | | | | | | | | | | | | | |
| --- | --- | --- | --- | --- | --- | --- | --- | --- | --- | --- | --- | --- | --- | --- |
|  | | **Result Code** | 1. Proceed with the interview  2. **Door lock**  **3. Refused**  4. Other specify.................................................................................. | | | | | | | | | | **Thank and end the**  **survey** | |
|  | | **Date and time of household visit (For volunteers) Date: ……../……../………Time:…….Am/Pm** | | | | | | | | | | | | |
|  | | **Health Block Name** | | | 1. Mao 2. Paomata 3. Oinam 4. Maram 5. Senapati HQ | | | If Mao, go to Q4 | | | | | | |
|  |  |  |  |  |  |  |  | If Paomata, go to Q5 | | | | | | |
|  |  |  |  |  |  |  |  | If Oinam, go to Q6 | | | | | | |
|  |  |  |  |  |  |  |  | If Maram, go to Q7 | | | | | | |
|  |  |  |  |  |  |  |  | If Senapati HQ, go to Q8 | | | | | | |
|  | | **(MAO Block )** Villages name | | | 1. Songsong 2. Kalinamai 3. Kayinu 4. Phikomai 5. Pudunamai 6. Chowainu 7. Robve Solephe 8. Punanamai | | | 1. Robunamai 2. Tadubi 3. Chakumai 4. Khridziiphi 5. Shajouba 6. Makhel 7. Tobumai 8. Kaibi | | | 1. Chawainamai Khullen 2. Chawainamai Khunou 3. Mao Pungdung 4. Emefiithu 5. Makhan Khullen 6. Makhan Centre 7. Makhan Lovadziizho 8. New Makhan | | | |
|  | | **(Paomata Block)** Villages name | | | 1. Paomata Centre 2. Makhufii 3. Tunggam Khullen 4. Tunggam Khunou 5. Tunggam Afii 6. Saranamai A 7. Saranamai B 8. Saranamai C 9. Kaikho 10. Rakhutao | | | 1. Tungjoy 2. Tungjoyv Upper 3. Tungjoy Lower 4. Tungjoy Middle 5. Tungjoy Rikhubumai 6. Liyai Khullen 7. Liyai Khunou 8. Liyai Chilao 9. Maiba 10. Phuba Khuman | | | 1. Phuba Thapham 2. Laii Village 3. Laii Shirafii 4. New Laii 5. Vaisiichu 6. Chingmai Khullen 7. Chingmai Khunou 8. Katafiimai | | | |
|  | | **(Oinam Block)** Villages name | | | 1. Oinam Hill 2. Ngamju (Upper Lower and) 3. Oinam Laila 4. Purul Akutpa 5. Purul Atongba 6. Purul Rosofii 7. Thingba Khunou 8. Chofii 9. Thingba Khullen 10. Koide Mathak 11. Koide Makha 12. Biisho | | | 1. Khamsom 2. Khamsom Huphii and Bazar 3. Keize 4. Reafii 5. Kapao 6. Tingsong Khullen 7. Tingsong Centre 8. Tingsong Khunou 9. Phaibung Khullen 10. Phaibung Khunou 11. Lower Phaibung 12. Lakhamai | | | 1. Shirong 2. Shirong Sofii 3. Kodom Khullen 4. Kodom Khavii 5. Khongdei Khuman 6. Khongdei Shimphung 7. Khongdei Ngawar 8. Ngari Khullen 9. Ngari Raidolumai 10. Ngari Lishang 11. Thiwa 12. Thiwa Songdo | | | |
|  | | **(Maram Block)** Villages name | | | 1. Maram Bazar 2. Makhan Khuman 3. Naojai 4. Maram Mathak Sagei 5. Maram Khullakpa Sagei 6. Maram Makha Sagei 7. New Magaimai 8. Mesue 9. Ramlung 10. New Maram 11. Patang 12. Kabinam 13. Kavanam 14. Maram Centre A 15. Maram Centre B 16. Maram Centre C 17. Sagongbam 18. Lairouching 19. Lairouching Nepali 20. Puni Pfosemai 21. Khongnem Thana 22. Maram Khongnem 23. Marafii 24. Karong 25. Vakho | | | 1. Sorbung 2. Taklung 3. Khabung Upper 4. Khabung Lower 5. Kathikho Karong 6. Mao Karong 7. Khabung Khunou 8. Ngatan 9. Maram Khunou 10. Katomei 11. Katomei centre 12. Katomei centre A (Nepali) 13. Natazang/saklim 14. Taphou Naga 15. Taphou Pudunamai 16. Taphou Nepali 17. Gurung Khutty 18. Sadim (Pukhri) 19. Malengli 20. Saragar 21. karingnam 22. Bangali 23. Gauthali 24. Mao Sadeni 25. Ragalong | | | 1. Tamphung 2. Chakha 3. Kangjang 4. Thuiyeng 5. Bakie 6. Bendramai 7. Zenemai 8. Zenamyi 9. Yangkhullen 10. Yangkhunou 11. Kenelu 12. Oklong 13. Oklong Khunou 14. Oklong Maryram 15. Makuilongdi 16. Rajaimai 17. langkungji 18. Kanem 19. Willong Khullen 20. Willong Khunou 21. Sangkhumei and 22. Sangkunglung 23. Kamalong 24. Pumdunlong | | | |
|  | | **(Senapati HQ block)** Villages name | | | 1. Senapati Village/TNK 2. Vielwand 3. Akailongdi 4. Zaikairii 5. Rikhumai Taphou 6. Mao Taphou 7. Poumai Taphou 8. Makhrielui 9. Taphou Phyamai | | | 1. Taphou Onaeme 2. Taphou Leangmai 3. Taphou Ngaihang 4. Emesiiphro 5. Senapati Bazar 6. Pouna Colony 7. Quarter Colony 8. Maralung 9. Katomei Makeng | | | 1. Makeng cheijinba 2. Takaimai 3. Council Colony 4. Namgailong Rongmai 5. New Eden 6. Zingsho Katomei 7. Taphou Namguigai & Namthang 8. Taphou Ngouningram 9. Taphou Matanmai & Marenmai village | | | |
|  | | Household No. | | | |  | | | | | | | |  |
|  | | Head of the family? | | 1. Yes 2. No   If yes, name of the head of the family..........................................................  Mobile No.: .................................................. | | | | | | | | | | If no, go to **Q18** |
|  | | **Example**  Block No. Village No.  Household No. Head of family    If other family member Individual ID.  Unique ID (Auto-generated)  **001**  **MAO**  MA0010001**H**01…..  **0001**  **H**  **M**  **System generated** | | | | | | | | | | | | |
|  | | Total family members | | | |  | | | | | | | |  |
|  | | Total family members who are not there at the time of screening, including living in some other districts/states/ nations | | | | Specify reasons.......................................... | | | | | | | |  |
|  | | On which month most of your household members are typically at home? | | | | 1. January 2. February 3. March 4. April 5. May | | 1. June 2. July 3. August 4. September 5. October | | | | 1. November 2. December 3. Almost every month | | |
|  | | On which day most of your household members are typically at home? | | | | 1. Monday 2. Tuesday 3. Wednesday | | 1. Thursday 2. Friday 3. Saturday | | | | 1. Sunday 2. Almost everyday | | |
|  | | At what time most of your household members are typically at home? | | | | 12am to 12pm........................... | | | | | | | |  |
|  | | What is the distance from your home to the nearest health facilities? (in Km) | | | | ..................km | | | | | | | |  |
|  | | Age (in years) | | | | Years | | | | | | | |  |
|  | | Gender | | | | 1. Male 2. Female 3. Other | | | | | | | |  |
|  | | Marital status | | | | 1. Married 2. Unmarried 3. Divorced/separated 4. Widow/widower | | | | | | | |  |
|  | | What is your highest level of education you have completed? | | | | 1. Illiterate 2. No formal school but can read and write 3. Upto primary level 4. Upto secondary level 5. Upto higher secondary level 6. Upto graduation/degree level 7. Master level and above | | | | | | | |  |
|  | | What is your occupation?  **(multiple answers)** | | | | 1. House wife 2. Student 3. Own cultivation 4. Agriculture labourer 5. Small business/petty/ tea shop 6. Livestock 7. Private 8. Government 9. Other (Specify)………………………………………….. | | | | | | | |  |
|  | | What is your monthly income (In Rs.)? | | | | 1. Up to 10000 2. 10001-20000 3. 20001-30000 4. 30001-40000 5. 40001-50000 6. 50001 and above 7. No income | | | | | | | |  |
|  | | Do you belong to the schedule tribe? | | | | Yes  If yes, specify tribe.......................... | No  If no, specify................................ | | | | | | |  |
| **Section:2 TB Knowledge** | | | | | | | | | | | | | | |
|  | | Have you heard of TB?(use local term for TB) | | | | Yes | No | | | | | | | If no, go to Q44 |
|  | | From where/whom did you hear?  **(Multiple answers)** | | | | 1. Doctor /other health worker at PHC /GH 2. TV 3. Radio 4. Newspaper 5. Posters/pamphlets 6. Health program 7. Self-help group meeting 8. Friends/peers 9. Family members/relatives 10. Neighbours 11. Self/TB history in the family 12. School/college education 13. Other specify……………………………… | | | | | | | |  |
|  | | Is TB Curable? | | | | 1. Yes 2. No 3. Don’t know | | | | | | | |  |
|  | | In your opinion who is prone to TB?  **(Multiple answers)** | | | | 1. Anyone 2. Women 3. Children 4. Men 5. Poor 6. Rich 7. Living in urban area 8. Living in rural area 9. Smokers 10. Alcohol user 11. Don’t know 12. Other specify………………………………. | | | | | | | |  |
|  | | Do you know how is TB caused?  **(Multiple answers)** | | | | 1. Micro-organisms 2. Hereditary 3. Malnutrition 4. Smoking/alcohol 5. Water 6. Dust 7. Don’t know 8. Other (specify)……………………………………… | | | | | | | |  |
|  | | How is TB Spread?  **(Multiple answers)** | | | | 1. Air 2. Food 3. Water 4. Don’t know 5. Other (specify)……………………………………………………… | | | | | | | |  |
|  | | What are the symptoms of TB?  **(Multiple answers)** | | | | 1. Cough that lasts longer than 2 weeks 2. Expectoration 3. Chest pain 4. Blood in sputum 5. Weight loss 6. Loss of appetite 7. Fever 8. Shortness of breath 9. Night sweats 10. Tiredness 11. Enlargement of neck glands(Lymph nodes) 12. Don’t know 13. Other(specify)..................................... | | | | | | | |  |
|  | | Do you know where is TB diagnosed?  **(Multiple answers)** | | | | 1. Govt. health facility 2. Private health facility 3. Health centers run by NGOs 4. Don’t know 5. Other (specify)………………………………… | | | | | | | |  |
|  | | Is treatment for TB available free of cost? | | | | 1. Yes 2. No 3. Don’t know | | | | | | | |  |
|  | | How can one prevent from getting TB?**(Multiple answers)** | | | | 1. Closing the mouth while coughing 2. Not spitting any where 3. Practicing good hygiene 4. Keeping the surroundings clean 5. Good nutrition 6. BCG vaccination 7. Don’t know 8. Other(specify)………………………………………… | | | | | | | |  |
| **Section:3 TB history [ (√) Tick appropriate box]** | | | | | | | | | | | | | | |
|  | Have you been diagnosed as TB-positive in the past five years? | | | | | Yes  If yes, Nikshay ID……………........ | | | No | | | | | Go to Q 44 |
|  | From which health facility you have been diagnosed as TB-positive? | | | | | 1. Govt. hospital 2. Private hospital 3. Health centers run by NGO 4. Other specify…………………………………………………… | | | | | | | |  |
|  | Have you taken anti-tuberculosis therapy (ATT) drug? | | | | | 1. Yes | | | 1. No   Reasons for not starting the treatment?.......................... | | | | | If no, go to,Q44 |
|  | Where was anti-tuberculosis therapy (ATT) initiated? | | | | | 1. Govt. hospital 2. Private hospital 3. Health centers run by NGO 4. Other specify………………………………………………………. | | | | | | | |  |
|  | When have you taken anti-tuberculosis therapy (ATT) drug? | | | | | 1. In the past 2. Taking currently | | | | | | | |  |
|  | What is the time period/duration taken from having a symptom to diagnosing a TB disease? (in days) | | | | | Days | | | | | | | |  |
|  | What is the time period/durations taken for initiating the treatment from diagnosing the disease (TB) to start the treatment? (in days ) | | | | | Days | | | | | | | |  |
|  | Have you taken/have been taking anti-tuberculosis therapy (ATT) drug regularly? | | | | | 1. Yes 2. No   If no, what is the reason? Specify……………………………………….. | | | | | | | |  |
|  | Have you completed treatment as per Doctor advised? | | | | | Yes  If yes, how long was the treatment duration? (in months)..................... | | No  If no, what are the reasons for not completing treatment?....................  …………………………………………… | | | | | |  |
| **Section: 4 TB symptom screening** | | | | | | | | | | | | | | |
|  | At present do you have any of the following symptoms? | | | | | | | | | | | | | If no, go to Q 60 |
|  | 1. Persistent cough for ≥2 weeks | | | | | Yes | | | | No | | | |  |
|  | 1. Fever for ≥2 weeks | | | | | Yes | | | | No | | | |  |
|  | 1. Loss of appetite | | | | | Yes | | | | No | | | |  |
|  | 1. Night sweats for more than 2 weeks or unintentional weight loss (loss of 4.5kg or >5kg of the usual body weight over a period of 6-12 months) | | | | | Yes | | | | No | | | |  |
|  | 1. Presence of blood in sputum any time during the last 6 months | | | | | Yes | | | | No | | | |  |
|  | 1. Chest pain in last one month | | | | | Yes | | | | No | | | |  |
| **Section: 5 Only for chest symptomatic persons** | | | | | | | | | | | | | | |
|  | Since how long you have been having the TB symptoms (in Weeks) | | | | | Week | | | | | | | |  |
|  | Have you visited any health facility for seeking care? | | | | | Yes | | | | No | | | | If no, go to Q59 |
|  | Which health facility did you access? | | | | | 1. Private hospital/doctors 2. Govt. health facility 3. Alt. system/homeo/ayur/sidha 4. Quack/Faith healing 5. Traditional healer 6. Others specify………………………………… | | | | | | | |  |
|  | Reason for choice of health facility | | | | | 1. Satisfaction with the health care provider 2. Quality of ease of getting care/waiting time 3. Quality of interpersonal/communication skills 4. low cost/payment for service 5. Quality of facility cleanliness 6. Referred 7. No other choice 8. Distance 9. Other specify…………………………………… | | | | | | | |  |
|  | What is the time interval between onset of symptoms and seeking care? | | | | | 1. Immediate 2. After week 3. One month 4. More than one month | | | | | | | |  |
|  | What action/treatment have you taken to cure the symptom | | | | | 1. Taken TB medicine given from visited hospital 2. Home remedies/native medicine 3. Medicines from pharmacy 4. Medicines frompetty/groceryshop 5. Visited Quack/Faith healing 6. Others specify………………………………… | | | | | | | |  |
|  | Average cost incurred for the diagnosis, treatment including travel, food & stay (Rs.) | | | | | Rs…………………………………………………………./- | | | | | | | |  |
|  | Do you face any problem in accessing existing health facilities?  **(multiple answers)** | | | | | 1. Nothing 2. Distance/Transport 3. Poor attitude of health care workers 4. lack of services 5. Absentee health care worker 6. Fee/money spent 7. Delay/long waiting time 8. Inconvenient timings 9. Others specify…………………………… | | | | | | | | Go to Q60 |
|  | Reasons for not seeking care  **(multiple answers)** | | | | | 1. Symptoms not severe enough 2. Pressure of work 3. Lack of money 4. Distance 5. Indifference 6. Dependence on alcohol/drugs 7. Dissatisfaction with health facility 8. Domestic/pre-occupation 9. Other specify……………………………… | | | | | | | |  |
| **Section:6 Individual Habits** | | | | | | | | | | | | | | |
|  | Do you have a habit of taking smokeless tobacco, such as (chewing tobacco, khaini, snuff, gutka, panmasala etc.) | | | | | 1. Never 2. Used in the past 3. Taking currently | | | | | | | |  |
|  | Do you have a habit of smoking? | | | | | 1. Never 2. Smoked in the past 3. Smoking currently | | | | | | | |  |
|  | Do you have a habit of drinking alcoholic products (such as locally prepared alcohol, beer, wine, whisky etc.) | | | | | 1. Never 2. Consume in the past 3. Taking currently | | | | | | | |  |
|  | Volunteer Name | | | | | ……………………………………………………………………………. | | | | | | | |  |

**Annexure-V**

**Second Round TB Symptom Screening Tool**

|  | Individual ID | | (Symstem Autogenerated ID) |  |
| --- | --- | --- | --- | --- |
|  | Date and time of household visit (For volunteers) | | DD_____MM____YY_______ TT______MM_____ |  |
|  | Result Code | | 1. Proceed with the interview  2. **Door lock**  **3. Refused**  4. Other specify................................................................. | **Thank and end the**  **survey** |
|  | **Section 1: TB symptom screening** | | | |
|  | At present do you have any of the following symptoms? | | | |
|  | 1. Persistent cough for ≥2 weeks | | Yes | No |
|  | 1. Fever for ≥2 weeks | | Yes | No |
|  | 1. Loss of appetite | | Yes | No |
|  | 1. Night sweats for more than 2 weeks or unintentional weight loss (loss of 4.5kg or >5kg of the usual body weight over a period of 6-12 months) | | Yes | No |
|  | 1. Presence of blood in sputum any time during the last 6 months | | Yes | No |
|  | 1. Chest pain in last one month | | Yes | No |
|  |  | |  |  |
|  |  | |  |  |
|  |  | |  |  |
|  |  | |  |  |
|  |  | |  |  |
| **Section 2: Individual Habits** | | | | |
|  | Do you have a habit of taking smokeless tobacco, such as (chewing tobacco, khaini, snuff, gutka, panmasala etc?) | 1. Never 2. Used in the past 3. Taking currently | |  |
|  | Do you have a habit of smoking? | 1. Never 2. Smoked in the past 3. Smoking currently | |  |
|  | Do you have a habit of drinking alcoholic products (such as locally prepared alcohol, beer, wine, whisky, etc?) | 1. Never 2. Consume in the past 3. Taking currently | |  |
|  | Volunteer Name | ……………………………………………………………………………. | |  |
|  | Form status | 1. Incomplete 2. Unverified 3. Complete | |  |

**Annexure VI**

**Fully confidential for Research**

**Part-I (After Initiation of Treatment)**

**National Institute for Research in Tuberculosis (ICMR)**

**Identifying costs contributing to catastrophic expenditure amongst TB patients registered under NTEP Senapati District, Manipur.**

## Interview Schedule

| Name of the interviewer |  | | | Codes for not interviewing | | |
| --- | --- | --- | --- | --- | --- | --- |
| Date of first attempt |  | | | 1=Not available 2=Died  3=Migrated 4=Too sick/hospitalized  5=Not traceable 6=Refused  7=Other (specify) | | |
| Date of second attempt |  | | |  |  |  |
| Date of third attempt |  | | |  |  |  |
| Reason for not interviewing | ------------------------------------- | | |  |  |  |
|  | | | | | | |
| Name & Address:  ­­­­­­­­­­ | | TB No | TU | | PHI | Year |
|  |  |  |  | |  |  |

| I. Clinical characteristics: (To be filled from TB treatment card) | | | | | | | | | | | | | |
| --- | --- | --- | --- | --- | --- | --- | --- | --- | --- | --- | --- | --- | --- |
|  | | Type of TB | | | | Pulmonary smear positive …………….  Pulmonary smear negative …………….  Extra-pulmonary …………………….. | | | | | | 1  2  3 |  |
|  | | Treatment Regimen | | | | Cat I (new Pulmonary) ………………...  Cat II (retreatment) ………………….... | | | | | | 1  2 |  |
|  | | Total duration of planned treatment | | | | 6 -Months……………………………  8 -Months…………………….……  Other (specify) ----------------------------- | | | | | | 1  2  3 |  |
|  | | HIV status | | | | Positive ………………………………..  Negative ……………………………….  Not tested ……………………………...  Unknown ……………………………... | | | | | | 1  2  3  4 |  |
| **II. General Information** | | | | | | | | | | | | | |
|  | Sex | | | | Male ……………………………… …..  Female …………………………………. | | | | | | 1  2 | |  |
|  | Age in years | | | |  | | | | | |  | |  |
|  | Education years of schooling | | | |  | | | | | |  | |  |
|  | Marital Status | | | | Unmarried ……………………………….  Married ………………………………….  Widowed ………………………………..  Separated ………………………………..  Divorced ………………………………... | | | | | | 1  2  3  4  5 | |  |
|  | Occupation | | | | Wage Earner …………………………….  Salary Earner ……………………………  Self Employed …………………………..  Housewife ………………………………  Student ………………………………….  Trainee ………………………………….  Unemployed …………………………….  Other (specify) ----------------------------- | | | | | | 1  2  3  4  5  6  7  8 | |  |
|  | Personal Income Rs. | | | | Day | | | | | |  | |  |
|  |  |  |  |  | Month | | | | | |  |  |  |
|  |  |  |  |  | Year | | | | | |  |  |  |
|  | Religion | | | | Hindu ……………………………………  Muslim ………………………………….  Christian ………………………………...  Other (specify) ----------------------------- | | | | | | 1  2  3  4 | |  |
|  | Community | | | | OC ………………………………………  BC ………………………………………  SC ………………………………………  ST ………………………………………. | | | | | | 1  2  3  4 | |  |
|  | Mother Tongue | | | | Tamil ……………………………………  Telugu …………………………………...  Hindi …………………………………….  Punjabi …………………………………..  Others specify …………………………... | | | | | | 1  2  3  4  5 | |  |
|  | Type of family | | | | Joint ………………………………….…..  Nuclear …………………………………. | | | | | | 1  2 | |  |
|  | Total members in the family | | | |  | | | | | |  | |  |
|  | No studied >10^th^Std in the family | | | |  | | | | | |  | |  |
|  | No of earning members in the family | | | |  | | | | | |  | |  |
|  | Family income per year | | | |  | | | | | |  | |  |
|  | Family food expenditure per month | | | |  | | | | | |  | |  |
|  | Duration of stay in years | | | |  | | | | | |  | |  |
| **III. Life style characteristics:** | | | | | | | | | | | | | |
|  |  | | | |  | | | Quantity consumed? | | |  | |  |
|  | **Chew paan masala** | | | | Yes..1  No...2 | | | 21. a How many pouch per day? | | | | |  |
|  | **Chew tobacco** | | | | Yes..1  No...2 | | | 22. a How many pouch per day? | | | | |  |
|  | **Smoking** | | | | Yes..1  No...2 | | | 23. a How many cigarette/beedi per day? | | | | |  |
|  | **Drink alcohol** | | | | Yes..1  No...2 | | | 24. a How much ml per week? | | | | |  |
|  | **Drugs** | | | | Yes..1  No...2 | | | 25. a How many per day? | | | | |  |
| **III. Previous Treatment** | | | | | | | | | | | | | |
|  | Have you ever had TB treatment before? | | | | Yes ….……………………………… …..  No …... …………………………………. | | | | | | 1  2 | |  |
|  | Have you completed treatment? | | | | Yes ….……………………………… …..  No …... …………………………………. | | | | | | 1  2 | | Go to 29 |
|  | Reasons for not complete | | | | Lack of money for treatment costs ……...  Drug side effects ………………………...  Moved …………………………………...  Distance to facility ………………………  Others specify …………………………... | | | | | | 1  2  3  4  5 | |  |
| **IV. Delay, Pre-diagnostic & Diagnostic Costs** | | | | | | | | | | | | | |
|  | What symptoms did you experience that led you to seek treatment for your current illness? | | | | | | **29. a**. How long did you experience these symptoms before you went to seek treatment | | | |  | |  |
|  |  | | Yes | No | | | In Days | | | |  |  |  |
|  | Cough | | 1 29.a | 2 | | |  | | | |  |  |  |
|  | Night sweats | | 1 29.a | 2 | | |  | | | |  |  |  |
|  | Coughing up blood | | 1 29.a | 2 | | |  | | | |  |  |  |
|  | Weight loss | | 1 29.a | 2 | | |  | | | |  |  |  |
|  | Fever | | 1 29.a | 2 | | |  | | | |  |  |  |
|  | Chest pain | | 1 29.a | 2 | | |  | | | |  |  |  |
|  | Other (specify) | | 1 29.a | 2 | | |  | | | |  |  |  |
|  | Where did you go first to seek treatment or advice for these symptoms | | | | **Govt:**  District hospital ………………………….  Dispensary ………………………………  Health Centre ……………………………  **Private:**  Mission hospital …………………………  Pharmacy, drug & grocery store ………...  Herbalist …………………………………  Private hospital/clinic …………………...  Other (specify) ----------------------------- | | | | | | 1  2  3  4  5  6  7  8 | | Go to 32 |
|  | Why did you not go to the public health facility, | | | | Distance to facility ………………………  Too expensive …………………………...  Time consuming to wait ………………...  Lack of available facilities ………………  Mistrust of government ………………….  Belief system ……………………………  No drugs available ………………………  Other (specify) ----------------------------- | | | | | | 1  2  3  4  5  6  7  8 | |  |
|  | Have you visited a traditional healer? | | | | Yes ….……………………………… …..  No …... …………………………………. | | | | | | 1  2 | |  |
|  | How far is the nearest government facility:   1. DMC: 2. Others: | | | | Distance in KM | | | | hours walking | hours with transport |  | |  |
|  |  |  |  |  |  | | | | . | . |  |  |  |
|  |  |  |  |  |  | | | | . | . |  |  |  |

1. **About how much did you spend for each of these visits before you were diagnosed with TB, including the visit when you actually received your diagnosis?**

|  | Date | Provider  Govt..1  Pvt….2  Others specify..3 | Registration & Consultation fees | Test  (for sputum  or others) | X-ray (includes travel & fees) | Drug costs  (all kinds total) | Travel | Food | Hospitalization  Yes. 1  No. 2 | Accommodation  Costs | Insurance Reimbursement | Work Absenteeism | Loss of income |
| --- | --- | --- | --- | --- | --- | --- | --- | --- | --- | --- | --- | --- | --- |
| **Diagnosis** |  |  |  |  |  |  |  |  |  |  |  |  |  |
| **Prior to Dx-1** |  |  |  |  |  |  |  |  |  |  |  |  |  |
| **Prior to Dx-2** |  |  |  |  |  |  |  |  |  |  |  |  |  |
| **Prior to Dx-3** |  |  |  |  |  |  |  |  |  |  |  |  |  |
| **Prior to Dx-4** |  |  |  |  |  |  |  |  |  |  |  |  |  |
| **Prior to Dx-5** |  |  |  |  |  |  |  |  |  |  |  |  |  |

| **V. Treatment Costs (***Costs related to DOT )* | | | | | |
| --- | --- | --- | --- | --- | --- |
|  | Where do you currently take your TB drugs? | Health facility / hospital ……………  Home ……………………………….  Community …………………………  Workplace ………………………….  Dispensary …………………………. | | 1  2  3  4  5 |  |
|  | How many times per week do you go to take your drugs? | 3 times ….…………………………..  5 times ……………………………...  6 times ……………………………...  Other (specify) -------------------------- | | 1  2  3  4 |  |
|  | How long does it take you to get there (one way) | hours walking | hours with transport |  |  |
|  |  | . | . |  |  |
|  | How long does one of these visits take on average, including time on the road and waiting time  (Total turnaround time)? | .hours | |  |  |
|  | From your home to the DOT place, how much does it cost if you take transport? (both ways) | Rs. | |  |  |
|  | How much do you spend on food on the road, while waiting, for lunch? | Rs. | |  |  |
|  |  |  | |  |  |

**VI. Information about house** (Circle appropriately)

0 2 4

1. Housing type (Katcha=0; Semipucca=2; Puccka=4) ……………………….

0 2

1. House (Rent=0; Own=2) ……………………………………………………………..

0 1

1. Separate kitchen in the house (No=0; Yes=1) …………………………………….

1 2 3

1. House flooring (Mud=1; Cement=2; Tiled=3) ……………………………….

0 1 2 4

1. Toilet facility (No facility/open place=0; shared /public pit=1;

shared flush=2; Own flush=4) …………………………………….

2 1 0

1. Source of lighting (Electric=2; Kerosene/gas/oil=1; Others=0) ……………

### VII. Information about essentials (Circle appropriately)

2 1 0

1. Source of drinking water (Pipe or hand pump or well in residence or

water purchased =2; tap or hand pump or well in public=1, Others=0) ………….

3 1 0

1. Main fuel for cooking (Electricity or Liquid petroleum gas or biogas=3;

Coal or charcoal or kerosene or fire wood purchased=1; Others=0) ……….

0 2 3 4

1. Ownership of agricultural land (No land=0; <2 acres or ……………..

acreage not known=2; 2-<5 acres=3; >5 acres=4)

0 2

1. Ownership of irrigated land (No irrigated land=0; Some irrigated land=2) ….

0 2

1. Ownership of livestock (No livestock=0; Owns livestock=2) …………………

### VIII. Ownership of durables (Circle appropriately) No Yes

0

4

1. Car …………………………………………………………..

0

4

1. Tractor ……………………………………………………….

0

3

1. Moped/Scooter/Motorcycle/Auto ………………………….

0

3

1. Telephone/Cell phone ……………………………………..

0

3

1. Refrigerator …………………………………………………

0

3

1. TV (color) …………………………………………………..
2. TV (B/W) …………………………………………………..

0

2

1. Air conditioner ……………………………………………..

0

3

0

3

1. Washings machine ………………………………………..

0

2

1. Bicycle ………………………………………………………

0

2

1. Electric fan ………………………………………………….

0

2

1. Radio/transistor/Tape recorder …………………………..

0

2

1. Sewing machine …………………………………………..

0

2

1. Water pump ……………………………………………….

0

2

1. Bullock cart ………………………………………………..

0

2

1. Thresher …………………………………………………..

0

1

1. Mattress/bed ………………………………………………

0

1

1. Pressure cooker/mixer/grinder ………………………….

0

1

1. Chair ………………………………………………………

0

1

1. Cot ………………………………………………………..

0

1

1. Table ……………………………………………………..

0

1

1. Clock/watch ………………………………………………

**X. Remarks:**

Name of investigator: Signature of investigator

Date:

**Annexure-VII**

**Fully confidential for Research**

**Part-II &III (At the end of Intensive phase/At the end of treatment)**

**National Institute for Research in Tuberculosis (ICMR)**

**Identifying costs contributing to catastrophic expenditure amongst TB patients registered under NTEP Senapati District, Manipur.**

## Interview Schedule

| Name of the interviewer |  | | | Codes for not interviewing | |
| --- | --- | --- | --- | --- | --- |
| Date of first attempt |  | | | 1=Not available  2=Died  3=Migrated  4=Too sick/hospitalized  5=Not traceable  6=Refused  7=Other (specify)  ____________________________ | |
| Date of second attempt |  | | |  |  |
| Date of third attempt |  | | |  |  |
| Reason for not interviewing |  | | |  |  |
|  | | | | | |
| Name & Address:  ­­­­­­­­­­ | | TB No | TU | PHI | Year |
|  |  |  |  |  |  |

| **I. Treatment Costs (*Costs related to DOT )*** | | | | |
| --- | --- | --- | --- | --- |
| 1 | Where do you currently take your TB drugs? | 1. Health facility / hospital  2. Community  3. Workplace  4. Dispensary | |  |
| 2 | How many times per week do you go to take your drugs? | 1. 3 times  2. 5 times  3. 6 times  4. Other (specify) _______________________ | |  |
| 3 | How long does it take you to get there (one way) | hours walking; | hours with transport; |  |
|  |  | _________  Minutes | ________  Minutes |  |
| 4 | How long does one of these visits take on average, including time on the road and waiting time  (Total turnaround time)? | _________ Minutes | |  |
| 5 | From your home to the DOT place, how much does it cost if you take transport? (both ways) | Rs. _________ | |  |
| 6 | How much do you spend on food on the road, while waiting, for lunch? | Rs. _________ | |  |

| **II. Costs related to picking up the TB drugs – where drugs are currently picked up** | | | |
| --- | --- | --- | --- |
| 7 | How often do you travel to the health facility / hospital for picking up your TB drugs? | _________ Times  _________ Months |  |
| 8 | How long does it take you to get there (one way)  hours walking  hours with transport | ________ Minutes  _________ Minutes |  |
| 9 | How long does one of these visits take on average, including time on the road and waiting time (total turnaround time)? | _________ Minutes |  |
| 10 | From your home to the facility, how much does it cost if you take transport? (both ways) | Rs. _________ |  |
| 11 | If you go to a facility to pick up your drugs, how much do you spend on food on that day? (On the road, while waiting, lunch etc.) | Rs. _________ |  |
| 12 | Do you have to pay administration fees when picking up your TB drugs? | 1. Yes  2. No | Go to 14 |
| 13 | If YES, how much? | Rs. _________ |  |
| 14 | Do you have any accommodation costs when picking up your TB drugs? | 1. Yes  2. No | Go to 16 |
| 15 | If YES: how much? | Rs. _________ |  |

| **III. Costs related to follow up tests** | | | | | | |
| --- | --- | --- | --- | --- | --- | --- |
| 16 | | Did you ever have to go to the health facility in addition to your regular visits for follow up tests since the beginning of treatment? | | 1. Yes  2. No | Go to 21 | |
| 17 | | If yes, how many times? | | _________ Times |  | |
| 18 | | If yes, did you have to pay any additional costs any time during the entire period? | | 1. Yes  2. No | Go to 20 | |
| 19 | | If so, what kind of costs and how much?  Fees  Sputum test  X-ray  TB Drugs  Other Drugs  Others | | Rs. _________  Rs. _________  Rs. _________  Rs. _________  Rs. _________  Rs. _________ |  | |
| 20 | | How long does one of these follow-up visits take on average, including time on the road, waiting time and tests (total turnaround time)? | | ________ Minutes |  | |
| **IV. Guardian Costs** | | | | | | |
| 21 | Does any family/friend/DOT supporter accompany you on any visits or go in your place to collect your TB drugs? | | 1. Yes  2. No | | | Go to 25 |
| 22 | *If YES,* on how many visits has your family/friend/DOT supporter accompanied you or gone in your place? *Record pre-diagnosis/diagnosis visits and treatment visits separately* | | ________ Diag. Times  ________ Treatment Times | | |  |
| 23 | How much does your friend/family/DOT supporter earn per day? | | 1. Rs. _________  2. Doesn’t earn | | |  |
| 24 | Why did someone accompany you? | | 1. Distance  2. Security  3. Administrative barriers 4. Too ill to travel alone  5. Was required for treatment 6. Other (specify)  __________________________ | | |  |

| **V. Hospitalization** | | | |
| --- | --- | --- | --- |
| 25 | Have you been hospitalized before or during your TB treatment? | 1. Yes  2. No | Go to 33 |
| 26 | If YES: how many days in total did you stay at the hospital? | ________ Days |  |
| 27 | How much did you pay in the hospital during your entire stay? | Hospital administration fees: Sheets/Linnen:  Food (not provided by hospital): Transport (return):  Drugs:  Tests:  Others: | Rs.___________  Rs. __________  Rs. __________  Rs. __________  Rs. __________  Rs. __________  Rs. __________ |
| 28 | Did any family/friend stay with you while in hospital? | 1. Yes  2. No | Go to 30 |
| 29 | If YES: How many days did he/she stay with you (sleep there)? | _______ Days |  |
| 30 | Were there any extra costs for your relative/friend for staying at the hospital? | 1. Yes  2. No | Go to 32 |
| 31 | IF Yes | Accommodation (hospital or other):Rs. ___________ Food: Rs. ___________  Transport: Rs. ___________ Other:Rs. ___________ |  |
| 32 | How much does your friend/family normally earn per day? | 1. Rs. _________  2. Doesn’t Earn |  |
| 33 | Did any other family/friend visit you while in hospital? | 1. Yes  2. No | Go to 36 |
| 34 | If yes, how many people visited you? | ________ Members |  |
| 35 | How many times did they visit you?  Accommodation per person: Food per person:  Transport per person: Other: | ________ Times  Rs. ___________  Rs. ___________  Rs. ___________  Rs. ___________ |  |
| 36 | How long were the visits including traveling time? | ________ Minutes |  |

| **VI. Other Costs Food Supplements** | | | |
| --- | --- | --- | --- |
| 37 | Do you buy any supplements for your diet because of the TB illness, for example vitamins, meat, energy drinks, soft drinks, fruits or medicines? | 1. Yes  2. No | Go to 40 |
| 38 | If YES: What kind of items? (specify)  1. Fruits  2. Drinks  3. Vitamins/Herbs  4. Meat  5. Other (specify): |  |  |
| 39 | How much did you spend on these items in the last month approximately? | Rs. _________ |  |

| **VII. Other Illnesses** | | | |
| --- | --- | --- | --- |
| 40 | Do you have any chronic illness for which you are receiving treatment? | 1. Yes  2. No | Go to 42 |
| 41 | If yes: which? | 1. Cardiac 2. Respiratory 3. Diabetics |  |
| 42 | Are there any additional costs for you because of this other illness besides the costs that you have already mentioned? | 1. Yes  2. No | Go to 44 |
| 43 | If YES: How much are these additional costs on average per month?  Tests:  Drugs:  Transport:  Food:  Other: | Total Amount: Rs. _________ |  |
| 44 | How much did you spend on healthcare on average per month BEFORE the TB illness?  How much did you spend on healthcare on average per month NOW? | Rs. _________  Rs. _________ |  |

| **VIII. Insurance** | | | |
| --- | --- | --- | --- |
| 45 | Do you have any kind of private or government health/medical insurance scheme? | 1. Yes  2. No | Go to 49 |
| 46 | If YES: What type? | 1. Govt. 2. Private |  |
| 47 | Have you received reimbursement for any costs related to the TB illness? | 1. Yes  2. No |  |
| 48 | How much have you received as reimbursement? | Rs. _________ |  |

| **IX.Coping Costs** | | | |
| --- | --- | --- | --- |
| 49 | Did you borrow any money to cover costs due to the TB illness? | 1. Yes  2. No | Go to 53 |
| 50 | If YES: How much did you borrow? | Rs. _________ |  |
| 51 | From whom did you borrow? | 1. Family  2. Neighbors/friends  3. Private bank  4. Cooperative  5. Other (specify): _________________________ |  |
| 52 | What is the interest rate on the loan? (%) | 1. _________%  2. I don’t pay any interest  3. I am not expected to pay back the money |  |
| 53 | Have you sold any of your property to finance the cost of the TB illness? | 1. Yes  2. No | Go to 57 |
| 54 | If YES: What did you sell? | 1. Land  2. Livestock  3. Transport/vehicle  4. Household item  5. Farm produce  6. Other (specify):  _________________________ |  |
| 55 | What is the estimated market value of the property you sold? | Rs. _________ |  |
| 56 | How much did you earn from the sale of your property? | Rs. _________ |  |

| **X.Socioeconomic Information Individual Situation and Income** | | | |
| --- | --- | --- | --- |
| 57 | Are you currently formally employed? | 1. Yes, formal work  2. No, informal work  3. On sick leave  4. Retired  5. School, university  6. Housework  7. Other (specify)  _____________________ |  |
| 58 | Is the reason for Not Working related to the TB illness? | 1. Yes  2. No | Go to 60 |
| 59 | If Yes: When was the last time you were working? | Date / Month; |  |
| 60 | How you are usually paid? | 1. cash  2. in kind  3. cash and in kind  4. not paid  5. bank transferred salary  6. other |  |
| 61 | What was your estimated personal take home earning per month BEFORE the TB illness? (includes welfare, disability, or other social support): | 1. Rs. _________ / per week  2. Don’t earn |  |
| 62 | What is your estimated personal take home earning per month NOW? (includes welfare, disability, or other social support) | 1. Rs. _________/ per week  2. Don’t earn |  |
| 63 | Is the change related to the TB illness? | 1. Yes  2. No |  |
| 64 | Have you ever stopped working/going to school/doing housework due to TB? | 1. Yes  2. No |  |
| 65 | If YES: for how long? | _________ Days |  |
| 66 | Does someone stay home specifically to take care of you? | 1. Yes  2. No | Go to 68 |
| 67 | If YES: for how long? | ________ Weeks |  |
| 68 | Did they quit their income-earning job to stay home and care for you? | 1. Yes  2. No |  |
| 69 | How regularly did you work before you became ill with TB? | 1. Throughout the year  2. Seasonal/part of the year  3. Day labor  4. Other |  |
| 70 | Did you have to change jobs when you became ill with TB? | 1. Yes  2. No |  |
| 71 | What is your main occupation? | 1. Daily wage  2. Salaried –Government  3. Salaried-Private  4. Self employed  5. Homemaker  6. Famer  7. Student  8. Aged  9.Other (specify) |  |
| 72 | How many hours did you work on average per day BEFORE you became ill with TB? | ________ Hours |  |
| 73 | How many hours do you work on average NOW per day? | ________ Hours |  |
| 74 | Is the change related to the TB illness? | 1. Yes  2. No |  |
| 75 | Is someone doing the work that you used to do? | 1. daughter  2. son  3. spouse  4. friend  5. nobody  6. other family |  |
| 76 | Do you have children of or below school age? | 1. Yes  2. No | Go to 78 |
| 77 | Do all of your children of school age attend school regularly? | 1. Yes  2. No |  |
| 78 | If NO: Why not? | 1. Needs to help around the house 2. No money for school fees  3. Also sick  4. Has to work to earn income  5. Other (specify): |  |
| 79 | Do any of your children of or below school age work to finance costs due to the TB illness? | 1. Yes  2. No |  |
| 80 | While you are sick if you employed someone to do the housework for your household, how much would you have to pay him/her per day? |  |  |
| 81 | While you are healthy if you employed someone to do the housework for your household, how much would you have to pay him/her per day? |  |  |
| 82 | Are you financially independent? | 1. Yes  2. No |  |
| 83 | If Yes: Has this resulted in a financial burden? | 1. Yes  2. No |  |
| 84 | Has the TB illness affected your social or private life in any way | 1. No  2. Divorce  3. Loss of Job  4. Dropped out of school  5. Separated from spouse/partner  6. disruption of sexual life  7. Sick child  8. Other (specify): |  |

| **XI. Household Income and Spending** | | | |
| --- | --- | --- | --- |
| 85 | How much do you estimate was the average income of your household per month BEFORE the TB illness? | 1. income patient:  2. income rest of household  3. welfare payments  4. government assistance  5. Other:  TOTAL: |  |
| 86 | How many of the household members are paid for working? | ________ Members |  |
| 87 | Besides yourself, does anyone else of your household receive treatment for TB? | 1. Yes  2. No |  |
| 88 | If Yes: How many? | ________ Members |  |
| 89 | How much food did your household consume every month on average BEFORE the TB illness? | Rs. _________ |  |
| 90 | How much food does your household consume NOW every month on average? | ________ Members |  |
| 91 | Has the amount of food consumed per month changed due to the TB illness? | 1. Yes  2. No |  |
| 92 | If the government could provide you with some service to ease the burden of TB on you and your household, what would you prefer to have? *State options, choose one* | 1. Transport vouchers  2. food vouchers  3. More efficient service  4. Other (specify): |  |

**XII. Remarks**

Name of investigator: Signature of investigator:

Date:
