# Supplementary material for: Breaking barriers for TB elimination: A novel community-led strategy revolutionizing tuberculosis case finding and treatment support in Senapati District Manipur-A quasi-experimental pre-post study protocol
Source: PLoS One. 2025 Jul 3;20(7):e0326324. doi: 10.1371/journal.pone.0326324 (PMC12225811; doi:10.1371/journal.pone.0326324)
Supplement: Attachment — Submitted filename: Peer Review-Manuscript PONE-D-24-38779.docx [file pone.0326324.s002.docx]

***Peer Review for MEDIN Manuscript PONE-D-24-38779***

*Date: 22-October-2024*

**“Breaking barriers for TB elimination: A novel community-led strategy revolutionizing Tuberculosis case finding and treatment support in Senapati District of Manipur-A quasi-experimental pre-post study protocol”**

These are my comments in the peer review for the manuscript requested.

***My general comments:***

In general, the authors presented a study protocol (a quasi-experimental pre-post study) for active case finding and treatment of tuberculosis (TB), focusing on inhabitants aged >15 years residing in the Senapati District, Manipur, India. This study aims to engage the Student and Women Organizations (SAWOs) in augmenting the TB case finding and treatment, based on clinical symptoms, chest xray and sputum testing (using rapid molecular test-CBNAAT). The rationale for this study is very good.

My first concern is that the active case finding (ACF) and treatment for TB elimination and engaging organizations to enhance the effectiveness of ACF is a novel idea. This has been performed and published by Australian Research Teams (Guy B.Marks, Greg J.Fox et al., <https://www.nejm.org/doi/full/10.1056/NEJMoa1902129>). My suggestion is that the authors should reconsider the word “*novel* community-led strategy revolutionizing…”. I think “*scaled up*” is better term.

My second concern is the significance of the gold standard for testing TB so that the comparison outcomes (TB case notification rate, adherence rate, and treatment outcome) between the intervention and control arms are more accurate. The rapid molecular test CBNAAT has been reported to have a sensitivity of 63% to 75% ((<https://pmc.ncbi.nlm.nih.gov/articles/PMC10654686/>), indicating that approximately 25% to 37% of TB cases would be missed if only using the rapid molecular test CBNAAT. From my clinical and research knowledge, there will be a certain proportion of TB patients with trace calls by molecular TB testing ( about 50%-60% reveals negative TB culture). I think that there should be included TB culture (MGIT), regarding the true TB population with false negative CBNAAT and/or normal chest xray.

I highly believe that the TB culture (MGIT method…), applied for negative TB molecular testing with clinical and radiological findings consistent with tuberculosis, will ensure that the overall outcome comparisons between intervention and control arms are best accurate.

***My specific comments:***

1. **Title:**

The title needs to be reconsidered, regarding the term “*novel* community-led strategy revolutionizing….”, because active case finding TB and treatment and engaging organizations is not a breakthrough.

2. **Abstract:** The abstract is clear, succinct, and well-written,
3. **Introduction:**

The introduction is very well written and clearly describes the TB burden in India and the rationale for this study to be conducted. The aims of this study have clearly been stated.

4. **Materials and Methods:**

The methods are well designed, structured and written.

4.1 I suggest that the authors think more about the number of TB patients with false negative rapid molecular test-CBNAAT (indeed, they have true TB infection) and trace calls from molecular testing (all estimated to account for 20%-30%) of all people tested with molecular testing. Further tuberculosis cultures (for example, with MGIT method) should be performed. This will ensure that the outcome comparisons between the intervention and control arms are accurate
4.2 I also think the authors should clarify the sampling method in village mapping and household enumeration (Lines 186 and 193). It should be randomly selected using a computerized generated sampling method in predetermining villages for intervention and standard-care arms. These factors are significant for preventing potential selection bias.
4.3 Regarding the study population, the exclusion of mobile population/visitors and institution populations (Lines 135-138) should be carefully considered. On the one hand, this will enhance survey completion and retention rates. On the other hand, these populations can further transmit the tuberculosis (in cases with undiagnosed active TB), moving from this village to other villages of the study sites. As a result, this will influence the primary study outcomes of TB notification rates and outcomes at a certain level.

From my personal view, I recommend including mobile populations, regarding my above consideration in balance with maximizing the retention rate in the study, generalizability, and optimizing the number of population receiving TB treatment and reducing cross-transmission. However, the authors should consider this point further.

5. **Discussion:** This is well supported and well written.

- **My final decision:** I think this is a great study and I support this study to be proceeded.
