# Supplementary material for: Breaking barriers for TB elimination: A novel community-led strategy revolutionizing tuberculosis case finding and treatment support in Senapati District Manipur-A quasi-experimental pre-post study protocol
Source: PLoS One. 2025 Jul 3;20(7):e0326324. doi: 10.1371/journal.pone.0326324 (PMC12225811; doi:10.1371/journal.pone.0326324)
Supplement: Attachment — Submitted filename: comment.docx [file pone.0326324.s003.docx]

This study is very important and the study is well designed. I have only minor comments below.

1. It is very important to instruct patients about how to collect quality sputum for the quality diagnosis but author did not mention it.
2. Out of two sputum collected (spot and early morning), which one is used for CBNAAT test?
