# Supplementary material for: Breaking barriers for TB elimination: A novel community-led strategy revolutionizing tuberculosis case finding and treatment support in Senapati District Manipur-A quasi-experimental pre-post study protocol
Source: PLoS One. 2025 Jul 3;20(7):e0326324. doi: 10.1371/journal.pone.0326324 (PMC12225811; doi:10.1371/journal.pone.0326324)
Supplement: Attachment — Submitted filename: 2. Response to Reviewers.docx [file pone.0326324.s005.docx]

**Response to Reviewers**

| **Sl. No.** | **Journal Requirements** | **Response/justifications** |
| --- | --- | --- |
| 1 | Please ensure that your manuscript meets PLOS ONE's style requirements, including those for file naming. The PLOS ONE style templates can be found at  https://journals.plos.org/plosone/s/file?id=wjVg/PLOSOne_formatting_sample_main_body.pdf and  https://journals.plos.org/plosone/s/file?id=ba62/PLOSOne_formatting_sample_title_authors_affiliations.pdf | Thank you for considering our manuscript; we have prepared the manuscript meeting PLOS ONE's style requirements, including those for file naming. |
| 2. | We note that you have indicated that there are restrictions to data sharing for this study. For studies involving human research participant data or other sensitive data, we encourage authors to share de-identified or anonymized data. However, when data cannot be publicly shared for ethical reasons, we allow authors to make their data sets available upon request. For information on unacceptable data access restrictions, please see <http://journals.plos.org/plosone/s/data-availability#loc-unacceptable-data-access-restrictions>. | This study aims to assess the impact of engaging Student and Women Organizations (SAWOs) in improving tuberculosis (TB) case notifications, treatment adherence and completion rates within the National Tuberculosis Elimination Program (NTEP), as well as estimate the cost-effectiveness of Active Case Finding (ACF) interventions. It is designed as a community-based participatory research initiative, addressing all four pillars of the ‘END TB’ strategy: Detect, Treat, Prevent and Build. The intervention includes activities such as community TB sensitization, ACF, treatment support and monitoring, using a quasi-experimental pre-post study design. Data will be collected on TB case notifications, treatment adherence and outcomes before and after the intervention.  As this is a study protocol, the aim of the protocol is to establish a solid foundation for the study’s implementation and ensure transparency in the proposed research methodology. Therefore, detailed data analysis or specific findings are not presented, as the manuscript primarily outlines the study’s methodology, objectives and design. The protocol provides a comprehensive description of the study framework, including intervention strategies, study phases, data collection methods and statistical approaches. The actual data will be collected and analyzed during the study’s execution and findings will be shared in subsequent publication(s) once the study is completed.  Since it is a protocol manuscript, we have not included any data while writing the original manuscript of this draft protocol, and accordingly, we declared the same as ‘Not Applicable’ under Data Availability section. |
| 3. | If there are ethical or legal restrictions on sharing a de-identified data set, please explain them in detail (e.g., data contain potentially identifying or sensitive patient information, data are owned by a third-party organization, etc.) and who has imposed them (e.g., a Research Ethics Committee or Institutional Review Board, etc.). Please also provide contact information for a data access committee, ethics committee, or other institutional body to which data requests may be sent. | There are certain restrictions on sharing data for our study as this involves sensitive data of TB patients.  However, if anyone is interested in our data, the de-identified data can be partially made available upon request, post-completion of the study.  The data can be made available post-completion of the study based on the request to   1. Dr. Harpreet Kaur   Email: [kaurh.hq@icmr.gov.in](mailto:kaurh.hq@icmr.gov.in)   2. Dr. Karikalan. N   Email: [karikalan.n@icmr.gov.in](mailto:karikalan.n@icmr.gov.in)   3. Dr. A. Stephen   Email: [stephen.a@icmr.gov.in](mailto:stephen.a@icmr.gov.in) |
| 4. | If there are no restrictions, please upload the minimal anonymized data set necessary to replicate your study findings to a stable, public repository and provide us with the relevant URLs, DOIs, or accession numbers. Please see <http://www.bmj.com/content/340/bmj.c181.long> for guidelines on how to de-identify and prepare clinical data for publication. For a list of recommended repositories, please see <https://journals.plos.org/plosone/s/recommended-repositories>. You also have the option of uploading the data as Supporting Information files, but we would recommend depositing data directly to a data repository if possible.  Please update your Data Availability statement in the submission form accordingly. | Since it is a protocol manuscript, we do not have the data to be shared at present. However, due to the sensitivity of the patient data, we would only be able to make available the de-identified data, upon request, post completion of the study. |
| 5. | Please amend either the abstract on the online submission form (via Edit Submission) or the abstract in the manuscript so that they are identical. | As suggested, we have checked/amended the abstract to be identical on both the online submission form and in the manuscript. |
| 6. | Please include a separate caption for each figure in your manuscript. | The figure we have provided has a separate caption and we have provided it in a separate Word file. |
| 7. | 5. Please review your reference list to ensure that it is complete and correct. If you have cited papers that have been retracted, please include the rationale for doing so in the manuscript text, or remove these references and replace them with relevant current references. Any changes to the reference list should be mentioned in the rebuttal letter that accompanies your revised manuscript. If you need to cite a retracted article, indicate the article’s retracted status in the References list and also include a citation and full reference for the retraction notice. | As suggested, we have checked the references and ensured to include complete and correct references. |
|  | **Reviewer #1:** |  |
| 1. | India remains the leading contributor to global burden of tuberculosis and it is obvious that in order to reduce global TB burden we need to reduce TB burden in India. At the same time in order to achieve END-TB goals in India we need to address TB diagnostics and treatment issues, including TB gaps in case finding, in every region and district in India, what will eventually result in reduction of incidence and mortality of TB in whole country. The manuscript provides lots of information and is a great example of national TB program activities in India. | We thank the Reviewer for acknowledging our work. |
| 2. | Are volunteers involved in active case finding provided with personal respiratory protection (FFP2/FFP3 respirators) since they might be exposed to lots of presumptive TB patients? | Yes, the volunteers engaged in active case finding are provided with respiratory masks (N95) to avoid exposure from presumptive TB cases.  Further, volunteers are also provided Personal Protective Equipment (PPE) Kits (face shield, glove, mask (N95), and hand sanitizer) to be used during screening and household visits for counseling the TB patients. Also, kits like vaccine carrier box, ice pack, falcon tube, parafilm M roll, tissue paper, sellotape, cotton, durable marker, and Ziplock cover were provided to the volunteers for collecting and transporting the sputum sample.  This aspect has been added in the revised manuscript, line numbers 451 to 456. |
| 3. | Does the protocol involve active contact tracing or it is not a part of the study, but has already been implemented by NTP in India? To clarify, there is and will be information on cases already registered in the district and contacts from those cases will be known to the NTP identifying certain households as focus of TB infection and they should be targeted for LTBI/TB diagnostics. Will that work be done within the proposed protocol? | Contact tracing activities (onsite diagnosis and treatment) are not done as a separate activity neither in this project nor under the NTEP program, since the volunteers are not trained in that. However, we do encourage, counsel and refer the household contacts of Pulmonary TB patients for further investigations & evaluations to rule out active TB to start the TB Preventive Treatment (TPT) as per the NTEP guidelines.    This aspect, again has been added in the revised manuscript, line numbers 284 to 295. |
| 4. | Following previous question – what are the follow-up actions if TB case, especially smear positive highly contagious, is identified throughout the study in the household? will the be a return visit for LTBI screening and possible LTBI treatment? | Once a confirmed TB case has been identified from the lab, the trained project staff informs / forwards the test result to the volunteer over phone to convey the test result to the patients. The volunteers visit the patient's house to inform the test result from whom the sputum is collected. With the consent of the patient, the volunteers also inform the test result to the other family members residing in the same household, apart from encouraging not only the family members to support the patient during their treatment but also provide counseling to the household contacts of pulmonary TB patients to go for further investigations & evaluations to rule out active TB to all the family members to start the TB Preventive Treatment as per the NTEP guideline.  Apart from that, the volunteers visit the patient's house at least once a week to monitor the treatment adherence from initiation to completion of the treatment.  When the patients do not adhere to the treatment, the volunteers do a counseling to complete the treatment.  The above has been added in the revised manuscript, line number 284 to 288. |
| 5. | Will rifampicin resistance/MDR be tested in cases throughout the study? Will treatment for DR TB be provided for those patients? | Yes, all diagnosed TB patients including rifampicin resistant / MDR cases are linked with the NTEP and also treated as per the Programme, as indicated in the revised manuscript, line numbers 287 and 295. |
|  | **Reviewer #2: My general comments:** |  |
| 1. | In general, the authors presented a study protocol (a quasi-experimental pre-post study) for active case finding and treatment of tuberculosis (TB), focusing on inhabitants aged >15 years residing in the Senapati District, Manipur, India. This study aims to engage the Student and Women Organizations (SAWOs) in augmenting the TB case finding and treatment, based on clinical symptoms, chest xray and sputum testing (using rapid molecular test-CBNAAT). The rationale for this study is very good. | We thank the Reviewer for encouraging words and acknowledging our work. |
| 2. | My first concern is that the active case finding (ACF) and treatment for TB elimination and engaging organizations to enhance the effectiveness of ACF is a novel idea. This has been performed and published by Australian Research Teams (Guy B.Marks, Greg J.Fox et al., <https://www.nejm.org/doi/full/10.1056/NEJMoa1902129>). My suggestion is that the authors should reconsider the word “novel community-led strategy revolutionizing…”. I think “scaled up” is better term. | We had used the term *novel* in the title, based on the existing literature, as we are involving the organized Student And Women Organizations (SAWOs) in TB case finding or in providing support during treatment. Though previous studies have engaged community members such as Community Healthcare Workers (CHWs), Accredited Social Health Activists (ASHAs), National Service Scheme (NSS) students and elementary and secondary school students, these individuals did not operate within a formal organizational structure that contributes to village-level decision-making on broader issues.  The SAWOs engaged in this project are well-structured organizations with clearly defined roles and responsibilities. They represent youth & women from their respective villages, communities, districts and collectively participate in larger forums to advance common goals and objectives. Leadership positions within these organizations are typically given to active and influential members of the group, chosen through an election process.  Each village, church and community (both tribal and non-tribal) has its own youth and women organizations. Members of student organizations include those who are currently pursuing their studies, or have either completed their education or have dropped out. However, most student leaders are individuals who have completed their education and are not currently employed.  Similarly, members of women organizations are primarily married women including widows, divorcees as well as those living with their husband and children.  This project is built on the four pillars of India's National Strategic plan that is Detect, treat, and prevent, Build. This project tries to address the cascade of care from educating people about TB to the identification of TB cases to the completion of the treatment.  This study brings a novel and comprehensive approach to addressing TB in the community by integrating SAWO engagement, cost-effectiveness analysis and a participatory research design. These elements set it apart from both NTEP’s ACF, which is more focused on diagnostic surveillance. By fostering community ownership, addressing all pillars of the END TB strategy and evaluating the economic impact, our study offers a holistic, inclusive and evaluative model for improving TB controls that could provide valuable insights for future TB programs.  In view of the foregoing rationale of engaging a unique & distinctly placed active and influential community group, operating from within a formal organizational structure, who are elected through a democratic process and contribute to village-level decision making on broader issues, and thus offer an opportunity for ownership of the intervention and some assurance of sustainability after project life, the authors think the study to be novel and thus request the Reviewer if novelty can be retained in the title of the manuscript, considering this group as a novel one, which is not the same as used in the referred publication by Australia research team. |
| 3. | My second concern is the significance of the gold standard for testing TB so that the comparison outcomes (TB case notification rate, adherence rate, and treatment outcome) between the intervention and control arms are more accurate. The rapid molecular test CBNAAT has been reported to have a sensitivity of 63% to 75% ((<https://pmc.ncbi.nlm.nih.gov/articles/PMC10654686/>), indicating that approximately 25% to 37% of TB cases would be missed if only using the rapid molecular test CBNAAT. From my clinical and research knowledge, there will be a certain proportion of TB patients with trace calls by molecular TB testing (about 50%-60% reveals negative TB culture). I think that there should be included TB culture (MGIT), regarding the true TB population with false negative CBNAAT and/or normal chest x-ray. | We agree with the Reviewer’s concern. However, the study district is a hard-to-reach area, and the testing is adopted as per recommendations of NTEP to use the molecular method as the first diagnostic tool to diagnose TB in this area, so that we can detect TB and DR-TB without delay. All our interventions have been implemented as per the routine NTEP activities except involving the volunteers from each village to carry out the activities.  As per the local TB program, CBNAAT has been used as the first diagnostic tool to diagnose the disease followed by chest X-Ray for those individuals whose sputum results turn out to be negative. Similarly, we are doing it in a programmatic mode to maintain the accuracy of our intervention (pre-and post).  The suggestion is 100% valid that we may miss approximately 25% to 37% of TB cases. However, adding culture (MGIT) will become a new intervention but the local NTEP is not doing it, and this may be considered as a limitation of the study, as this will further delay the study completion, which is both time bound and financially constrained. We will however recommend this to NETP to add culture / MGIT and make it program inclusive. |
| 4. | I highly believe that the TB culture (MGIT method…), applied for negative TB molecular testing with clinical and radiological findings consistent with tuberculosis, will ensure that the overall outcome comparisons between intervention and control arms are best accurate. | Though we are not doing culture for those CBNAAT-negative samples as per the local TB program. However, we are referring those individuals whose sputum turns out to be negative in the CBNAAT test to the nearest chest X-Ray centre for further investigations, as mentioned in the revised manuscript, line numbers 289 to 295. |
|  | **Reviewer #2: My specific comments:** |  |
| 1. | **Title:** The title needs to be reconsidered, regarding the term “novel community-led strategy revolutionizing….”, because active case finding TB and treatment and engaging organizations is not a breakthrough. | We had used the term *novel* in the title, based on the existing literature, as we are involving the organized Student And Women Organizations (SAWOs) in TB case finding or in providing support during treatment. Though previous studies have engaged community members such as Community Healthcare Workers (CHWs), Accredited Social Health Activists (ASHAs), National Service Scheme (NSS) students and elementary and secondary school students, these individuals did not operate within a formal organizational structure that contributes to village-level decision-making on broader issues.  The SAWOs engaged in this project are well-structured organizations with clearly defined roles and responsibilities. They represent youth & women from their respective villages, communities, districts and collectively participate in larger forums to advance common goals and objectives. Leadership positions within these organizations are typically given to active and influential members of the group, chosen through an election process.  Each village, church and community (both tribal and non-tribal) has its own youth and women organizations. Members of student organizations include those who are currently pursuing their studies, or have either completed their education or have dropped out. However, most student leaders are individuals who have completed their education and are not currently employed.  Similarly, members of women organizations are primarily married women including widows, divorcees as well as those living with their husband and children.  This project is built on the four pillars of India's National Strategic plan that is Detect, treat, and prevent, Build. This project tries to address the cascade of care from educating people about TB to the identification of TB cases to the completion of the treatment.  This study brings a novel and comprehensive approach to addressing TB in the community by integrating SAWO engagement, cost-effectiveness analysis and a participatory research design. These elements set it apart from both NTEP’s ACF, which is more focused on diagnostic surveillance. By fostering community ownership, addressing all pillars of the END TB strategy and evaluating the economic impact, our study offers a holistic, inclusive and evaluative model for improving TB controls that could provide valuable insights for future TB programs.  In view of the foregoing rationale of engaging a unique & distinctly placed active and influential community group, operating from within a formal organizational structure, who are elected through a democratic process and contribute to village-level decision making on broader issues, and thus offer an opportunity for ownership of the intervention and some assurance of sustainability after project life, the authors think the study to be novel and thus request the Reviewer if novelty can be retained in the title of the manuscript, considering this group as a novel one, which is not the same as used in the referred publication by Australia research team. |
| 2. | **Abstract:** The abstract is clear, succinct, and well-written, | We thank the Reviewer for encouraging words |
| 3. | **Introduction:** The introduction is very well written and clearly describes the TB burden in India and the rationale for this study to be conducted. The aims of this study have clearly been stated. | We thank the Reviewer for acknowledging our work. |
| 4. | **Material & Methods:** The methods are well designed, structured and written. | We thank the Reviewer for appreciating our work. |
| 4.1 | I suggest that the authors think more about the number of TB patients with false negative rapid molecular test-CBNAAT (indeed, they have true TB infection) and trace calls from molecular testing (all estimated to account for 20%-30%) of all people tested with molecular testing. Further tuberculosis cultures (for example, with MGIT method) should be performed.  This will ensure that the outcome comparisons between the intervention and control arms are accurate | We agree with the Reviewer’s concern. However, the study district is a hard-to-reach area, and the testing is adopted as per recommendations of NTEP to use the molecular method as the first diagnostic tool to diagnose TB in this area, so that we can detect TB and DR-TB without delay. All our interventions have been implemented as per the routine NTEP activities except involving the volunteers from each village to carry out the activities.  As per the local TB program, CBNAAT has been used as the first diagnostic tool to diagnose the disease followed by chest X-Ray for those individuals whose sputum results turn out to be negative. Similarly, we are doing it in a programmatic mode to maintain the accuracy of our intervention (pre-and post).  The suggestion is 100% valid that we may miss approximately 25% to 37% of TB cases. However, adding culture (MGIT) will become a new intervention but the local NTEP is not doing it, and this may be considered as limitation of the study, as this will further delay the study completion, which is both time bound and financially constraint. We will however recommend this to NETP to add culture / MGIT and make it program inclusive.under ‘My General Comments’. |
| 4.2 | I also think the authors should clarify the sampling method in village mapping and household enumeration (Lines 186 and 193). It should be randomly selected using a computerized generated sampling method in predetermining villages for intervention and standard-care arms. These factors are significant for preventing potential selection bias. | Our study is a pre-post study design, which involves screening of all the villages of the study district (as highlighted in line numbers 206 -213) village mapping is being carried out for the whole villages of the district, and household enumeration is done based on the study eligibility criteria for the whole district population. Hence there is no selection bias in the sampling method in village mapping and household enumeration. |
| 4.3 | Regarding the study population, the exclusion of mobile population/visitors and institution populations (Lines 135-138) should be carefully considered. On the one hand, this will enhance survey completion and retention rates. On the other hand, these populations can further transmit the tuberculosis (in cases with undiagnosed active TB), moving from this village to other villages of the study sites. As a result, this will influence the primary study outcomes of TB notification rates and outcomes at a certain level.  From my personal view, I recommend including mobile populations, regarding my above consideration in balance with maximizing the retention rate in the study, generalizability, and optimizing the number of population receiving TB treatment and reducing cross- transmission. However, the authors should consider this point further. | Since our project covers all the villages of Senapati district, intra-district migration from one village to another in the same district (i.e., within district migration) issues have been taken care and we are able to include such mobile populations.  Further, based on our enumeration and 1^st^ round of screening experiences even after excluding the mobile population, we came across a huge population difference in the enumerated population and the number of people who have been screened during the 1^st^ round of screening. We have randomly selected a few villages to cross verify the reasons for not being able to be screened in the 1^st^ round of screening after being included during the enumeration. We found that a lot of people migrate to other districts and states for work/studies. However, we cannot capture such mobile populations, which move from the study district to another district/state and this becomes limitation of the study as this is beyond the scope of our study.  On the other hand, we could consider including the institution populations like schools, colleges and offices as screening is done early morning and evening. We have made the changes accordingly in our study inclusion criteria in the revised manuscript, line numbers 151-155. |
| 5. | **Discussion:** The discussion is supported and well written. | We thank the reviewer for appreciating our work |
| 6. | **Decision:** I think this is a great study and I support this study to be proceeded. | We thank the reviewer for supporting for our work. |
|  | **Reviewer #3** |  |
| 1. | In this study protocol, the authors intend to study the impact of student and women organizations in improving TB case notifications. India has committed to end TB by 2025, this research seems to be late whereas such an intervention should have been a part of the program. | We understand that we have nearly approached the target of India’s commitment of ‘end TB’ by 2025. However research and new interventions are being emphasized to achieve this goal in a pragmatic way.  While the TB program in India has started ACF activities along with routine PCF activities across the country, there are some districts and states of India that were not able to implement ACF activities, especially in tribal & hard-to-reach areas, with Senapati district being one such area. For this reason, our project is being implemented as a demonstration model project as a part of those areas where ACF is not happening. |
| 2. | The protocol paper comes at a time when the sample collection is nearing completion. | Though the study has started and the first round of screening has progressed, we are yet to complete most of the work of the second round of screening and data collections. |
| 3. | Notwithstanding the above, as far as the protocol is concerned, there are a few issues that the authors need to clarify and address: | As under. |
| 3a | Details of the district including the health infrastructure both public and private, community-based organizations working for TB etc need to be elaborated for the reader to have an understanding the necessity of the intervention. | As per the suggestion, we have included ‘Health Infrastructure’ details in the revised manuscript line numbers 117 to 120. |
| 3b | In the data from national program shared in Table 1 on TB notifications, adherence and outcomes it is observed that the program has achieved >100% of the target. It is strange that the authors mention they by this intervention achieve 80% success in the targets. The fact that the program has achieved more than the research proposes makes this study redundant. | While national program data indicate high notification rates, our study focuses on assessing sustained and realistic programmatic success at the ground level. The reported >100% achievement reflects aggregated targets but gaps remain in treatment adherence, loss to follow-up and long-term success rates. Our intervention specifically addresses these gaps, ensuring feasibility within a real-world setting. Rather than being redundant, it may be worthwhile to consider that the study validates targeted, locally adapted strategies that could enhance the national program’s effectiveness. |
| 3c | The exclusion of the highly vulnerable populations such as mobile population/visitors, institutional populations (schools, colleges, offices, prisons, defence establishments, hospitals, nursing homes, hostels) and areas where survey operations are considered not to be feasible like insecurity areas is not clearly explained as large numbers of TB cases can be missed by such exclusion. The aim is to achieve a “comprehensive” approach to TB elimination. | Since our project covers all the villages of Senapati district, intra-district migration from one village to another in the same district (i.e., within district migration) issues have been taken care and we are able to include such mobile populations.  Further, based on our enumeration and 1^st^ round of screening experiences even after excluding the mobile population, we came across a huge population difference in the enumerated population and the number of people who have been screened during the 1^st^ round of screening. We have randomly selected a few villages to cross verify the reasons for not being able to be screened in the 1^st^ round of screening after being included during the enumeration. We found that a lot of people migrate to other districts and states for work/studies. However, we cannot capture such mobile populations, which move from the study district to another district/state and this becomes limitation of the study as this is beyond the scope of our study.  On the other hand, we could consider including the institution populations like schools, colleges and offices as the screening is done early morning and evening. We have made the changes accordingly in our study inclusion criteria in the revised manuscript, line numbers 151-155. |
| 3d. | Additionally, the authors have restricted the term “comprehensive approach” to the cascade of TB care whereas “comprehensive” would entail involvement of ALL stakeholders in the TB cascade such as private health care providers, NGOs/CBOs, TB champions etc. | This study, which comprehensively addresses all pillars of TB control as per the National Strategic Plan 2017-2025, also adopts a comprehensive approach in involving ALL stakeholders in the TB cascade project is a comprehensive approach, whereby we have involved the Community Based Organizations (CBOs) like Senapati District Student Association (SDSA) and Senapati District Women Association (SDWA) alongwith their federating units (Sub units), and also involved the TB Champions (survivors) in our project as they are members of SDSA, SDWA and their federating units. Further, we thought of also involving the private health care providers and the NGOs in our project, however, we found that there were no private hospitals in the district and there were no NGOs currently working in the district on tuberculosis. |
| 3e. | Baseline assessment need to include anthropometric and nutrition data which are the current focus of all TB programs. | Anthropometric and nutrition data are the current focus of TB programs. However, considering the feasibility of collecting all these details along with our activities within a short period of time, with resource constraints, we have not included this in our study.  The suggestion is very valid and we could consider the same in the upcoming project(s). |
| 3f. | As per recommendation after symptom screening, X-ray screening comes next and then microbiological examination. This especially in a community setting and with the recent emphasis on asymptomatic sub clinical TB. Linkages with the NTEP need to be elaborated in more detail in line 250. | As per the local TB program, upfront CBNAAT has been used as the diagnostic tool to diagnose the TB disease followed by chest X-Ray for those individuals, whose two sputum (on spot and early morning samples) results turn out to be negative. Similarly, we are doing it in a program way to maintain uniformity (Pre and post-intervention).  Regarding the linkages with the NTEP, we have added more details in the revised manuscript, lines numbers 277 to 288, as per the suggestions. |
| 3g. | Since the study is nearing completion, it would be good to provide interim data on achievement so far. | Since this is a study protocol paper, we will include the study results in this manuscript later in separate manuscript(s). |
| 3h. | Lines 340, 341 mentioning direct costs, the authors mention doctor’s consultation fees, x-ray and sputum exam expenses - Where does this come in? cases are directly referred to NTEP! | Though the TB cases are directly linked to the NTEP for the treatment. Some patients could refuse to take treatment under the TB program.  Our project tries to retain a maximum number of patients under the TB program after being diagnosed for their treatment. So, in this project, we would like to see how many patients we were able to retain under the TB program throughout their treatment and how many patients we lost to the private practitioners for their treatment as compared to the usual TB program.  One of the end TB goals is “no patients to incur catastrophic cost” and it was reported that ACF is one of the strategies to provide financial protection to patients. We try to estimate the patient cost incurred by the patient for diagnosis and treatment. This detail will be collected from the patients who have been diagnosed and treated under the TB program.  Also, we will collect data from both the patients who have been enrolled under our intervention and the usual TB program patients regarding the amount of money that they have spent for diagnosis and treatment in terms of doctor’s consultation, investigation fees and the drug cost in case, if any patient have been charged. These details are also being collected to estimate the cost-effectiveness of our intervention. |
| 3i. | Line 421, there is a mention of barriers to access to health care – was this studied in the study area what were the findings, if not this should be part of the study protocol. | In manuscript line number 421 which is now line no 478, what we mean to convey is that by involving SAWOs, the study introduces an approach that could be more sustainable and effective in reaching underserved populations and hard-to-reach areas because the volunteers are from the same locality/village/community and they have deep-rooted connections within the community, which can help overcome barriers related to demography, language, trust and access to healthcare services. |
| 3j. | Line 422, authors mention that the study addresses the social and behavioural factors that influence disease transmission as well as treatment outcomes by involving community members as active participants in TB control activities. What were these factors the authors wish to study? Were TB champions also involved in the process? | In manuscript line No.478 earlier 422, what we mean to convey is that, since the local CBOs like SDSA, SDWA, and their sub-unit organizations have taken an active role from educating the people to screening TB symptoms from door-to-door to monitoring and providing support to the patients during their treatment until the patients have completed their treatment. Their role involves addressing a lot of social and behavioral related issues like educating the people about TB (causes, transmissions, risk factors, precautions to be taken and treatment procedures). Also, their role involved addressing the stigma attached to the disease within the family and communities.  Since TB champions are part of the members of SDSA, SDWA and their sub-unit organizations, they are also part of the team. |
| 3k. | Line 435, the authors mention about the comprehensive approach. However, what they describe is the cascade of care and not a comprehensive approach which would entail involving all stakeholders in the process of ACF as mentioned above. Authors can refer to the many publications in the Indian context. | This study, which comprehensively addresses all pillars of TB control as per the National Strategic Plan 2017-2025, also adopts a comprehensive approach in involving ALL stakeholders in the TB care cascade. This project is a comprehensive approach, whereby we have involved the Community Based Organizations (CBOs) like Senapati District Student Association (SDSA) and Senapati District Women Association (SDWA) alongwith their federating units (Sub units), and also involved the TB Champions (survivors) in our project as they are members of SDSA, SDWA and their federating units. Further, we thought of also involving the private health care providers and the NGOs in our project, however, we found that there were no private hospitals in the district and there were no NGOs currently working in the district on tuberculosis.  The publications that the authors have referred to, in the Indian context, have been provided in references 18-21 and 27-35. |
|  | **Reviewer #4:** |  |
| 1. | The objectives of the study protocol are clearly stated and ethically sound. Description of the research stages well described. | We thank the Reviewer for encouraging words |
| 2. | Would it be possible to mention the population size (15 y.o and above) of the Senapati district as according to the protocol approximately 316 volunteers will be involved to the activities ( 1 person per 150 households; 47,411 households in total) and later how many people were reached by volunteers, please? | Population size/detail of 15 years and above is not available as the study district has been bifurcated into two districts (Senapati & kangpokpi) in 2016 by the Government of Manipur. Moreover, since 2021 census data has not been published by the Government of India, we do not have any secondary documents (baseline data) to refer to.  We have added whatever secondary data we got from the 2011 census (before bifurcation) and the district NTEP report data in the revised manuscript line numbers 101 to 115.  Regarding the number of populations covered by the volunteers, the same will be made available in different publications, as the work is still ongoing. |
| 3. | On the implementation of the ACF phase, I would suggest to write more about Infection control measures for volunteers while making the screening, collecting sputum samples and packing specimens for the transportation to the laboratory. How the transportation of the specimen will be organized (who transport, how, the distance to the nearest CB-NAAT point) | As per the reviewer suggestions, the information related to infection control along with the procedure has been added in the revised manuscript, line numbers 443-456  Personal Protective Equipment (PPE) Kits (face shield, glove, mask, hand sanitizer etc.) will be provided to the volunteers to be used during screening or household visits for counseling the TB patients. Also, kits like vaccine carrier box, ice pack, falcon tube, parafilm M roll, tissue paper, cellotape, cotton, durable marker, ziplock cover etc. will also be provided to the volunteers for collecting and transporting the sputum samples. All the kits will be handed over to the volunteers after completing rigorous two-day hands-on training.  Also, we have added sample collection and transportation details in the revised version of manuscript line numbers 273 to 277 as per the reviewer suggestions |
| 4. | Although the study is ongoing, it is a second round of the ACF, I am looking forward seeing the results of the study! | The study results will be published in the upcoming manuscript once the study is completed. Since this is a protocol manuscript, we felt it is better not to include the study results. |
|  | **Reviewer #5:** |  |
| 1. | The novelty of the concept may appear limited, as community systems for TB case finding have been previously documented in countries such as Ethiopia, South Africa, and Kenya, which the authors may wish to reference. Additionally, patient support group initiatives in Kerala bear some resemblance to this proposal, though these are more specifically targeted towards diagnosed patients. | Though the community systems for TB case finding have been previously documented, we did not find any study involving organized student and women organizations (SAWOs) in TB case finding or in providing support during the treatment. Even the previously documented studies from other countries such as Ethiopia, South Africa, Kenya, and even Kerala from India, as referred to by the Reviewer have documented engagement of community members such as Community Healthcare Workers (CHWs), Accredited Social Health Activists (ASHAs), National Service Scheme (NSS) students and elementary and secondary school students etc., these individuals did not operate within a formal organizational structure that contributes to village-level decision-making on broader issues.  The SAWOs engaged in this project are well-structured organizations with clearly defined roles and responsibilities. They represent youth & women from their respective villages, communities, districts and collectively participate in larger forums to advance common goals and objectives. Leadership positions within these organizations are typically given to active and influential members of the group, chosen through an election process.  Each village, church and community (both tribal and non-tribal) has its own youth and women organizations. Members of student organizations include those who are currently pursuing their studies, or have either completed their education or have dropped out. However, most student leaders are individuals who have completed their education and are not currently employed.  Similarly, members of women organizations are primarily married women including widows, divorcees as well as those living with their husband and children.  In view of the foregoing rationale of engaging a unique & distinctly placed active and influential community group, operating from within a formal organizational structure, who are elected through a democratic process and contribute to village-level decision making on broader issues, and thus offer an opportunity for ownership of the intervention and some assurance of sustainability after project life, the authors think the concept of the study to be novel, considering this group as a novel one.  This project is built on the four pillars of India's National Strategic Plan that is Detect, treat, and prevent, Build. This project tries to address the cascade of care from educating people about TB to the identification of TB cases to the completion of the treatment.  This study brings a novel and comprehensive approach to addressing TB in the community by integrating SAWO engagement, cost-effectiveness analysis and a participatory research design. These elements set it apart from both NTEP’s ACF, which is more focused on diagnostic surveillance. By fostering community ownership, addressing all pillars of the END TB strategy and evaluating the economic impact, our study offers a holistic, inclusive and evaluative model for improving TB controls that could provide valuable insights for future TB programs. |
| 2. | A key contribution of this research could lie in its quantification of community systems—a measurement that has not yet been fully developed. | Thank you for highlighting the potential contribution of our research in quantifying community systems, an area that remains underexplored. |
| 3. | The authors should also consider providing a rationale for the target adjustments made by the National TB Program (NTP) over the years. This will support accurate impact estimation, as target adjustments are often subjectively set. Collaborating with WHO and the NTP team could help establish a reliable baseline, as solely relying on NTP targets might lead to skewed analysis. | We are already collaborating with the state NTEP and Central TB Division and we are happy to collaborate with the WHO. As suggested, we will discuss with the WHO consultants for further suggestions. |
|  | **Reviewer #6:** |  |
| 1. | This study is important highest TB burden country and the study is well designed and recommend to accept for publication. | We thank the Reviewer for recognizing the importance of our study, appreciating its design and for recommending the paper for publication. |
|  | **Reviewer #7:** |  |
| 1 | Methodology section is incomplete, CRF components to be mentioned in the methodology section | The information which we are collecting during the first and second rounds has been added under the Methodology Section in the manuscript line numbers 239 to 245, as per the Reviewer’s suggestion. |
| 2. | Difference between ACF by NTEP and ECF executed by this project to be mentioned in detail. Also the uniqueness and novelty of the study in terms of community engagement and its difference from the existing community outreach programs by NMC to be mentioned in detail | We are carrying out both activities, ACF (as per the NTEP Program) as well as ECF. Our intervention difference is the engagement of volunteers to carry out the activities to improve TB case notifications, treatment adherence and completion rates as compared to existing routine strategies under the NTEP.  Though the community systems for TB case finding have been previously documented, we did not find any study involving organized student and women organizations (SAWOs) in TB case finding or in providing support during the treatment. Even the previously documented studies from other countries such as Ethiopia, South Africa, Kenya, and even Kerala from India have documented engagement of community members such as Community Healthcare Workers (CHWs), Accredited Social Health Activists (ASHAs), National Service Scheme (NSS) students and elementary and secondary school students etc., these individuals did not operate within a formal organizational structure that contributes to village-level decision-making on broader issues.  The SAWOs engaged in this project are well-structured organizations with clearly defined roles and responsibilities. They represent youth & women from their respective villages, communities, districts and collectively participate in larger forums to advance common goals and objectives. Leadership positions within these organizations are typically given to active and influential members of the group, chosen through an election process.  Each village, church and community (both tribal and non-tribal) has its own youth and women organizations. Members of student organizations include those who are currently pursuing their studies, or have either completed their education or have dropped out. However, most student leaders are individuals who have completed their education and are not currently employed.  Similarly, members of women organizations are primarily married women including widows, divorcees as well as those living with their husband and children.  In view of the foregoing rationale of engaging a unique & distinctly placed active and influential community group, operating from within a formal organizational structure, who are elected through a democratic process and contribute to village-level decision making on broader issues, and thus offer an opportunity for ownership of the intervention and some assurance of sustainability after project life, the authors think the concept of the study to be novel, considering this group as a novel one.  This project is built on the four pillars of India's National Strategic plan that is Detect, treat, and prevent, Build. This project tries to address the cascade of care from educating people about TB to the identification of TB cases to the completion of the treatment.  This study brings a novel and comprehensive approach to addressing TB in the community by integrating SAWO engagement, cost-effectiveness analysis and a participatory research design. These elements set it apart from both NTEP’s ACF, which is more focused on diagnostic surveillance, and NMC’s outreach programs, which are more clinical and education-based. By fostering community ownership, addressing all pillars of the END TB strategy and evaluating the economic impact, our study offers a holistic, inclusive and evaluative model for improving TB control that could provide valuable insights for future TB programs. |
| 3. | Target population to be defined precisely | Our target population is Individuals >15 years of age residing in the Senapati District of Manipur for at least a minimum of one month or more. We plan to cover the whole district population as per our study inclusion criteria. |
| 4. | Any similar studies done in other countries or other programs may be quoted as reference | Provided in reference 18-21 and 27-35. |
|  | **Reviewer #8 (Major Comments):** |  |
| 1. | Page 3, Line 33-36: The introduction needs better contextualization of the TB burden in Manipur. Specific local challenges could be highlighted in more detail, beyond the general statement about India's TB burden. This would strengthen the rationale for the study. | The needful has been done in the revised manuscript line numbers 60 - 74 |
| 2. | Page 4, Line 77-84: The study timeline is mentioned to be extended by 6 months due to disruptions, but the impact of these disruptions on study outcomes is not fully discussed. Please elaborate on how these events might affect the findings, participant retention, or data collection. | Since this is a study protocol, the exact impact of the disruptions on study outcomes, participant retention and data collection can only be fully assessed once the study is completed. However, we anticipate that the extended timeline may influence factors such as participant follow-up, adherence to treatment, and volunteer drop-out. The disruptions could potentially lead to delays in participant recruitment or loss to follow-up, which might affect the timing of data collection and overall study outcomes.  We do understand that these disruptions have likely adverse impacts on the above aspects of the study but this is something that is beyond our control. Once the study is completed, a thorough analysis will however be conducted to evaluate how these disruptions have impacted the results, and appropriate adjustments will be considered in the final interpretation of the findings. |
| 3. | Page 5, Line 107-110: The TB burden in Senapati district is noted as unknown, yet a similar neighboring district's TB burden is cited. Consider providing more data or clarifying why exact prevalence estimates are unavailable. This would support the choice of study site and methodology. | We have provided the TB prevalence data of the neighboring district (having a similar setting) because no TB prevalence data was available for the Senapati district where the study is being implemented as no TB prevalence survey has been conducted in our study district as per our knowledge. |
| 4. | Page 15, Line 373-374: The methodology lacks detail on how potential confounders will be identified and controlled in the logistic regression analysis. Please specify the strategies for confounder identification and handling, as this is crucial for the robustness of the results. | The individual level patient data will be obtained from the Nikshay portal. The potential confounders from the patient level and facility level data based on contextual factors such as facility size, patient volume, or baseline performance will be identified based on expert opinion and bivariate analysis. To control for confounding, we will include the confounders in the multi-level logistic regression model. Sensitivity analyses will also be conducted to assess the robustness of our findings. The same have been added on line numbers 414-423. |
|  | **Reviewer #8 (Minor Comments):** |  |
| 1. | Page 2: A more detailed definition of the role of Student and Women Organizations (SAWOs) would benefit readers unfamiliar with these groups. | We have added more details about the SAWOs in the revised manuscript lines numbers 68 to 82 as per the reviewer suggestions. |
| 2. | Page 7, Line 158: Typo in 'volunteers give written consent' - 'give' should be 'gave'. Please correct verb tense consistency | Corrected as per the reviewer suggestions |
|  | **Reviewer #9** |  |
| 1. | The protocol does indeed present a novel approach to TB case finding and overall TB control in Senapati District of Manipur, India. The use of student and women local organizations offers an opportunity for ownership of the intervention by the community and some assurance of sustainability after project life | We thank the Reviewer for acknowledging our work. |
| 2. | The protocol is presented soundly and manages to put across activities that will carried out during project life. However, the authors could consider the following to make the protocol clearer and avoid confusion with the readers; For example, line 196-198 talks about village mapping and assigning of unique identification numbers to participants according to the designated area. It will be helpful for the authors to describe and show how this process will be implemented | The Unique Individual Identification Number (UIIN), which is assigned to participants in the study, is generated using REDCap software (electronic data capture tool), which is installed into the volunteer’s mobile devices during the training session for implementation.  This detail has been added under the subheading *Household enumeration* in the revised manuscript line numbers 215 to 227, as per the Reviewer’s suggestion. |
| 3. | Line 215-217 talks about screening tools…the authors should state that a symptom screening tool will be used other and state any other tool will be used if any | We felt it may be more appropriate to retain it as a screening tool instead of a symptom screening tool because the screening tool not only includes TB symptoms but also other details like basic socio-demographic details, TB knowledge, TB history, individual habits of taking tobacco & alcohol as well as health-seeking behavior of people who have TB symptoms.  For this, we have added more details on what is included in first and second-round screening tools in manuscript line numbers 241 to 245.  Apart from the 1^st^ and 2^nd^ round of screening tools, no other tool will be used to collect the data. |
| 4. | Line 393-399, the authors describe payments/stipends that will be given to the volunteers. • An honorarium of ₹500 per month • mobile recharge of ₹700 per quarter • NTEP schemes NTEP; informant incentive (₹500/- Patient support incentive; ₹1000 for drug sensitive TB patients and ₹5000 for MDR-TB It would helpful for the authors to provide context and justification for these payments and how they circle back to sustainability after project life. | Under this project, we are providing an additional honorarium of ₹500 per month and ₹700 per quarter for the mobile recharge, apart from the NTEP scheme to encourage the volunteers to join the activities. We are trying this model in a trial mode with additional incentives apart from the usual NTEP schemes.  If our project model turns out to be effective and cost-efficient, we would like to recommend our model to the policymakers to integrate the same into a program mode so that the volunteers could continue to do the service with the same financial benefits being provided.  Even without additional financial benefits post completion of our project, the NTEP has some few schemes (informant incentive ₹500/- per patient, Patient support incentive; ₹1000 for drug-sensitive TB patients and ₹5000 for MDR-TB) that the volunteers can avail and continue to do their service.  All this information has been delivered to the volunteers while getting consent from them before starting their training sessions. Page numbers 443 -450. |
| 5. | Overall, this protocol could benefit from a copy editor to make readability and flow of the protocol better. | Edited as per the Reviewer suggestions |
|  | **Reviewer #10:** |  |
| 1. | The manuscript describes an interesting approach to TB case finding in a vulnerable and a violence prone area in East India. The effort to undertake it is laudable. | We thank the Reviewer for acknowledging our research work. |
| 2. | At the onset it would be desirable to know why 64% of TB symptomatics do not seek treatment. The Project should aim to elicit some responses from the TB cases detected in the study to guide mitigation strategies in the location context. | 64% of TB symptomatic do not seek treatment is part of the findings of India National TB Prevalence Project. The reasons were ignoring the symptoms (68%), not recognizing the symptoms as TB (18%), self-treatment (12%), and couldn’t afford to seek care (2%).  Since our project also captures the health-seeking behavior of people who have TB symptoms, we could consider the suggestions provided by the Reviewer. |
| 3. | The method section needs to elaborate on the approach to handling of asymptomatics between the 2 phases of intervention | Between the two rounds of interventions, our activities are only limited to unscheduled screening, which is defined as unplanned screening, where the community people (people who have TB symptoms) approach the volunteers to be screened for TB symptoms anytime post-completion of the active door-to-door screening. For the asymptomatic cases of household contacts of pulmonary TB patients, the volunteers are encouraging and counseling the household contacts of pulmonary TB patients to go for further investigations and evaluations at the nearest CHC/PHI/PHC/DTC to rule out active TB to start the TB Preventive Treatment (TPT), as per the NTEP guidelines.  For asymptomatic cases, we are not able to do much, apart from the referral service to the nearest CHC/PHI/PHC/DTC because the volunteers are not trained in this field. |
| 4. | It is unclear as to who has designed the costing tool and how is the costing data going to be analyzed. The researcher team needs to be well trained in eliciting responses to costing questions which would be best undertaken in the paper format. | Health economics expert from ICMR-NIRT has designed the costing tool and has provided rigorous training to the project staff before proceeding with the data collection in RedCap software, as our team is well trained in such a format.  Our cost analysis plan is provided in both the original and revised versions of the manuscript line numbers 384 to 390. |
| 5. | The methods section also needs to describe the deployment of human resources for each household and estimate the time taken to complete the activities for identification of TB cases at the household level. Would be useful for future studies using a similar approach. | For the deployment of human resources (volunteers) for screening and follow-up, we have allocated one volunteer per 150 household.  This detail has been provided in manuscript line numbers 190 to 200 and 235 to 250. |
| 6. | It should be clarified as to what were the selection criteria if any for the SAWO volunteers | The eligibility to become a volunteer has been provided in manuscript line numbers 182 to 188. |
| 7. | The interim time period between the 2 intervention phases of 6 months appears on the lower side. A period of 12 months may have been more productive. In between the SAWO volunteers could be engaged in community health education activities to supplement case finding. | Initially, our intervention was designed for 12 months. However, based on the subject expert comment considering the time and resource constraints, we had to shorten our intervention period from 12 months to 6 months. Although, as rightly pointed out, 12 months could have been more productive. |
| 8. | Fig.1 is indicative of good outcome indices. Would the present initiative be able to better these outcomes significantly. If any other foreseen tangible benefits to the community are identified, it would be helpful to mention them. | Some of the foreseen tangible benefits to the community are empowering them in health decision-making, early diagnosis, early initiation of treatment, better adherence and treatment outcome. Also, the community will benefit by the education being provided during our TB awareness campaign on the topics like the burden of TB, causes of TB, mode of transmission, preventive measures, treatment procedures, misconception of the disease etc. All these benefits have been provided in the manuscript.  Detailed benefits to the community will be part of our study findings, so any other tangible benefit will be added in our upcoming manuscript(s). |
| 9. | Reasons for refusal to participate in the survey should also be recorded and analysed. We look forward to the results arising from this approach which maybe further strengthened by observations on its sustainability. | Reasons for refusal to participate in the survey are being recorded and will be analyzed. |
| 10. | In the section on exposure to risk factors (line 118), the level of under nutrition in the community needs mentioning in view of the low SES and the high prevalence of TB. | We did not find any secondary literature on the nutritional status of adults in the study district of Senapati. Hence, it is not mentioned in the manuscript. |
| 11. | Taking as an example the questionnaire on TB KAP Section 2 and 3 words like "drug resistant TB", virus, bacteria, fungi (Section 2) and "public health problem" (section 3) should be replaced in a way that is understood by the local population. | All the questionnaires were interpreted by the trained project staff and the trained volunteers in local dialects. So, we are confident that the volunteers and the local population will not find any difficulty in understanding the terms given in our questionnaire. |
|  | **Anonymous Reviewers** |  |
| 1. | This study is very important and the study is well designed. I have only minor comments below.  1. It is very important to instruct patients about how to collect quality sputum for the quality diagnosis but author did not mention it. | We thank the Reviewer for acknowledging our research work.  Regarding the quality sputum collections, we have provided adequate instructions and trained the volunteers during the two-day hands-on training sessions organized on how to extract quality sputum and take care of the several aspects of collecting the sputum samples, which are as follows:   1. To go to open space/well-ventilated areas. 2. To make sure the falcon tube is clean before use and not to open it until the tube is ready for use. 3. To label the tube before giving it to the presumptive TB individuals. 4. To rinse the mouth with water before collecting samples. 5. To let the presumptive TB individuals, take a deep breath and hold the air for 5 seconds, and to slowly breathe out. Take another deep breath and cough hard until some sputum comes up (phlegm), not just saliva. 6. To collect samples not less than 5 ml. 7. To let the presumptive TB individuals, screw the cap on the cup tightly so it doesn’t leak. 8. To pack the samples as per NTEP guidelines and transport on the same day to the nearest health center (PHC/PHI/CHC/DTC).   Since the volunteers will be with the individuals who will be providing sputum samples, we are confident that they can help in quality diagnosis. |
| 2. | Out of two sputum collected (spot and early morning), which one is used for CBNAAT  test? | First, we use early morning samples for the test. If the morning sample result turns out to be negative, we run for the second time on the ‘spot’ sample to cross verify. If any sample result turns out to be positive, we consider it as a positive TB case.  If both the samples (on spot and early morning) are tested negative in the molecular test, such an individual with the negative result is again referred to the nearest X-Ray center for further investigations & evaluations. |
